# Adaptive conjunctive cognitive training (ACCT) in virtual reality for chronic stroke patients: a randomized controlled pilot trial

**DOI:** 10.1186/s12984-020-0652-3

**Published:** 2020-03-06

**Authors:** Martina Maier, Belén Rubio Ballester, Nuria Leiva Bañuelos, Esther Duarte Oller, Paul F. M. J. Verschure

**Affiliations:** 1 Laboratory of Synthetic, Perceptive, Emotive and Cognitive Systems (SPECS), Institute for Bioengineering of Catalonia (IBEC), The Barcelona Institute of Science and Technology, Av. d’Eduard Maristany 10-14, 08930 Barcelona, Spain; 2 Rehabilitation Research Group, Institut Hospital del Mar d’Investigacions Mèdiques (IMIM), Physical Medicine and Rehabilitation Department Parc de Salut Mar (Hospital del Mar, Hospital de l’Esperança), Barcelona, Spain; 3 Institució Catalana de Recerca I Estudis Avançats (ICREA), Barcelona, Spain

**Keywords:** Stroke, Rehabilitation, Cognitive deficits, Depression, Virtual reality

## Abstract

**Background:**

Current evidence for the effectiveness of post-stroke cognitive rehabilitation is weak, possibly due to two reasons. First, patients typically express cognitive deficits in several domains. Therapies focusing on specific cognitive deficits might not address their interrelated neurological nature. Second, co-occurring psychological problems are often neglected or not diagnosed, although post-stroke depression is common and related to cognitive deficits. This pilot trial aims to test a rehabilitation program in virtual reality that trains various cognitive domains in conjunction, by adapting to the patient’s disability and while investigating the influence of comorbidities.

**Methods:**

Thirty community-dwelling stroke patients at the chronic stage and suffering from cognitive impairment performed 30 min of daily training for 6 weeks. The experimental group followed, so called, adaptive conjunctive cognitive training (ACCT) using RGS, whereas the control group solved standard cognitive tasks at home for an equivalent amount of time. A comprehensive test battery covering executive function, spatial awareness, attention, and memory as well as independence, depression, and motor impairment was applied at baseline, at 6 weeks and 18-weeks follow-up.

**Results:**

At baseline, 75% of our sample had an impairment in more than one cognitive domain. The experimental group showed improvements in attention (${\chi}_F^2$ (2) = 9.57, *p* < .01), spatial awareness (${\chi}_F^2$ (2) = 11.23, *p* < .01) and generalized cognitive functioning (${\chi}_F^2$ (2) = 15.5, *p* < .001). No significant change was seen in the executive function and memory domain. For the control group, no significant change over time was found. Further, they worsened in their depression level after treatment (*T* = 45, *r* = .72, *p* < .01) but returned to baseline at follow-up. The experimental group displayed a lower level of depression than the control group after treatment (*Ws* = 81.5, z = − 2.76, *r* = − .60, *p* < .01) and (*Ws* = 92, z = − 2.03, *r* = − .44, *p* < .05).

**Conclusions:**

ACCT positively influences attention and spatial awareness, as well as depressive mood in chronic stroke patients.

**Trial registration:**

The trial was registered prospectively at ClinicalTrials.gov (NCT02816008) on June 21, 2016.

## Background

Cognitive impairments are common after stroke, with incident rates up to 78% [1]. Patients with mild cognitive impairment are at risk for developing dementia [2]. Cognitive deficits correlate with poor functional outcomes and increased risk of dependence [3], have negative effects on the patient’s quality of life [4], and alter the patient’s ability to socialize [5]. However, the current clinical practice seems to lack methods that specifically address cognitive sequelae. According to a meta-analysis that aimed at proposing recommendations for new clinical standards, currently available treatments that are used as control conditions are conventional therapies like physical therapy or occupational therapy, pseudo treatments like mental or social stimulation without therapeutic intent, as well as psychosocial interventions like psychotherapy or emotional support for individuals or groups [6]. Besides, it has been shown that cognitively impaired patients participate less in rehabilitation activities, which potentially contributes to the poorer functional outcome they display [7]. Finding effective cognitive rehabilitation methods that can be incorporated in clinical practice is therefore crucial. Numerous methods to improve cognitive deficits, for instance, specifically attention [8], memory [9], executive function [10], or spatial abilities [11], have been proposed. However, the results show mixed efficacies. A meta-analysis on the impact of attentional treatments showed an effect on divided attention in the short-term, but found no evidence for persisting effects on other attentional domains, global attention, or functional outcomes [12]. Similarly, a meta-review that investigated the effect of memory rehabilitation found that training might benefit subjective reports of memory in the short term, but shows no effect in the long term, on objective memory measures, mood, functional abilities or quality of life [13]. Ultimately, a meta-analysis over 6 Cochrane reviews shows insufficient research evidence or evidence of insufficient quality to support any recommendation for cognitive stroke rehabilitation [14]. Besides methodological issues, one limitation of existing methods could be that they focus on one deficit only, ignoring that patients typically express deficits in multiple cognitive domains [1, 2]. A study on a large sample of heterogeneous stroke patients which aimed at linking lesions to cognitive deficits found that a given lesion location leads to cognitive impairments in several domains [15]. This emphasizes that cognitive functions rely on a network of brain regions. A lesion in one of those regions might cause a disturbance to the network, which leads to a multitude of symptoms. This is further supported by studies that revealed that pathological changes in brain structures are related to the occurrence of various cognitive deficits and symptoms for instance, in Alzheimer’s disease [16] or spatial neglect [17]. Moreover, the presence of multiple cognitive deficits seems to be a marker in patients that are at risk of developing Alzheimer’s disease later in life [18]. To what extent rehabilitation could potentially drive structural or functional changes to alleviate the symptoms of stroke is still under debate [19, 20]. Nevertheless, rehabilitation methods have to aid the patient in obtaining enough functionality to independently perform instrumental activities of daily living, be it through restoration of function or compensation. With this in mind, focusing on training a single cognitive skill might not be efficient because many daily tasks or jobs require several cognitive abilities for their execution [21]. For instance, most patients would like to be mobile and drive a car again after their stroke. Driving requires the individual to use selective attention to deal with the traffic, traffic signs and distractions, to be cognitively flexible to react to changing situations on the road, to visually scan the mirrors at the front, at the side, and in the back, to have a visual field that includes the sidewalks and to perform all of this while steering the car effectively in real-time [22]. Consequently, rehabilitation methods that address one specific cognitive ability only do not address the requirements of performing the activities of daily living and might not stimulate and train the underlying brain processes adequately. If a stroke leads to impairments in various cognitive domains, then these domains should be treated together to benefit a patient’s performance in everyday life.

To address the challenge of simultaneously training various cognitive abilities in an individualized manner, we revert to interactive technologies, in particular to the coupling of motion capture technology with virtual reality (VR). VR-based systems have shown to be at least as effective as conventional therapies for physical rehabilitation, such as for the recovery of upper limb movements [23–25] or gait and balance [26]. Contrarily, meta-analyses investigating the use of VR for stroke rehabilitation were either not able to analyze the effect of training on cognitive function [25] or only found a preliminary positive effect [24] due to insufficient randomized controlled trials. Besides, computer-based interventions for cognitive rehabilitation are currently only recommended as a practice option when supervised by a therapist [27]. The positive effect of VR for physical recovery, however, is only confirmed for those systems that incorporate distinct neuroscientific and psychological principles that underlie learning and recovery [23, 28]. It appears that cognitive rehabilitation methods can also include principles of learning, like repetitive practice, increasing difficulty or complexity and providing feedback through auditory or verbal cues [29, 30]. However, it seems that these principles are either not explicitly declared in the interventions, or the field still needs to evaluate the exact mechanisms behind cognitive rehabilitation that would positively alter cognitive function and behaviour [14]. This leads to the paradoxical situation, that although many cognitive rehabilitation protocols rely on technology (18 out of 44 studies in the meta-analyses mentioned here [10–12, 31]), VR appears to be rarely used in cognitive rehabilitation (4 studies in [29]). More specifically, certain principles of neurorehabilitation can be better implemented in virtual than in physical reality. For instance, a recent study has shown that the intention compatible enhancement of movement is beneficial in counteracting learned non-use [32]. This enhancement is only possible when the properties of visual feedback are manipulated beyond the properties of the physical world. There are indications that such enhanced feedback can be used in cognitive rehabilitation too. Some rehabilitation methods for reducing spatial neglect use VR to recreate realistic scenarios (e.g., crossroads) that allow the patients to train attentional abilities in an ecologically valid but safe environment [29]. Augmented visual or auditory feedback provides them with a more enriched and controlled learning situation than reality would be able to offer [33]. The VR system used in the current study combines specifically two principles of neurorehabilitation: increasing and individualizing difficulty as well as embodied first-person practice [28]. The principle of increasing difficulty is grounded on the finding that learning is maximal if a task is individualized to the subject and provides training at an optimal challenge level [34, 35]. This principle was also advanced as being beneficial for cognitive rehabilitation [36]. A study that provided computerized working memory training which increased the difficulty level of each training task automatically to the patient’s working memory capacity found a significant improvement in trained and untrained working memory tasks [9], which is similar to another study where the difficulty adapted as a function of individual performance and where feedback was provided through scores and verbal encouragement [37]. Indeed, in VR, we can create tasks that require the patient to use abilities from various cognitive domains to achieve a given goal [38]. Algorithms can learn from the patient’s performance and adapt the difficulty of the task gradually and automatically to identify the current ability level of the patient and to challenge it appropriately [39], potentially allowing a heterogeneous group of cognitively impaired individuals to train in a consistent rehabilitation regime. The principle of embodied practice relies on the insights gained from the studies of action observation [40]. It is also the primary rationale behind the Rehabilitation Gaming System (RGS) [41, 42], a VR rehabilitation tool on which the development of the training program presented here is based. RGS promotes functional recovery at all stages post-stroke [43] and cortical reorganization [44] through an integrated approach that combines action execution and observation [45] with goal-oriented integrated tasks. In RGS the patient controls an avatar on a computer screen and observes the avatar’s movement from a first-person perspective. This embodied training could benefit cognitive rehabilitation too, as motor and cognitive skills training contributes to activity changes in common brain regions [46]. Indeed, earlier theoretical work has shown that we can also think of the motor system as forming an integral part of cognitive control systems [47, 48]. Besides delivering individualized, embodied and immersive training, using a VR-based system might also promote motivation through presenting complex goal-oriented tasks combined with gamification [49]. Patients identified the lack of motivation as one of the factors preventing them from completing post-stroke exercise programs [50]. Lack of adherence appears to be a known issue in cognitive rehabilitation as well [51]. However, the exact relationship between adherence and motivation as well as the factors which in turn define and affect internal states need to be investigated. Ultimately, VR-based systems are apt to increase training time and intensity and can extend the training to the patient’s home after discharge from the hospital [44], as they operate in an automated fashion, require less personnel, and are more cost-effective than traditional rehabilitation methods [52]. It is, therefore, worthwhile to investigate the effectiveness of science and evidence-based VR systems for cognitive recovery as they can overcome current limitations in cognitive rehabilitation, such as labour-intensiveness, isolated treatment of cognitive deficits and missing knowledge of the active ingredients in treatments [14].

Another issue in cognitive rehabilitation is that co-occurring post-stroke depression is often not detected [53]. However, depression is common after stroke—although incident rates can vary substantially between studies, pooled frequency is estimated to be at 31% [54]. Patients with post-stroke depression show lower cognitive functioning as well as a higher dependency in activities of daily living, more severe impairments, and handicap than non-depressed patients [55]. Poor performance in neuropsychological tests, therefore, can be attributed not only to stroke, age [56] and the inefficacy of cognitive training but also mood disorders. On the other hand, cognitive rehabilitation can influence depressive mood positively, as shown in patients with mild cognitive impairment [57]. Thus, the presence of depression should be measured in cognitively impaired patients, and its interaction with cognitive functioning and cognitive rehabilitation should be investigated when patients with cognitive deficits are treated.

Here, we propose and test a novel method for the conjunctive training of cognitive abilities from multiple cognitive domains. We developed integrated cognitive rehabilitation scenarios in VR to address deficits in memory, attention, spatial awareness, and executive function in combination and in a task- and goal-oriented manner. This proposal reflects the fundamental consideration that specific cognitive abilities are constituent aspects of cognition rather than isolated domains or, in other words, processes that are critically linked in the overall architecture of the brain [58]. The implementation of these scenarios includes a mechanism that adapts the difficulty automatically to the patient’s capabilities using machine learning techniques [59], thereby addressing unique profiles of impairments and skills in a heterogeneous group of stroke patients. The algorithm adapts several task parameters, which reflect cognitive abilities, to the performance of the patient and hence adjusts the task’s difficulty automatically. The task parameters fitting the user’s performance provide a user-specific model. The development of the adaptive conjunctive cognitive training (ACCT) program studied here is based on the existing VR rehabilitation tool RGS, which provides a task-oriented and gamified training from a first-person embodied perspective through an avatar immersed in multi-modal task environments [41, 42]. This explorative pilot study aims to identify potential effects and challenges in anticipation of a larger trial. We compare the ACCT intervention against a control group that performs a standard at-home cognitive rehabilitation program. We hypothesize that the training scenarios can adapt the difficulty to the individual cognitive impairment level of each patient, equalizing performance differences. Further, we expect to see that the ACCT intervention positively influences the patient’s impairment level in the four cognitive domains addressed. Knowing that observed effects could be potentially modulated by post-stroke depression, we also analyze in a subgroup whether depression negatively influences cognitive functioning and can be positively modulated by the ACCT intervention.

## Methods

### Study design and patients

We conducted a randomized controlled pilot trial with an intended allocation ratio of 1:1, which was approved by the local Ethical Committee at Parc de Salut Mar and registered at ClinicalTrials.gov (NCT02816008). Recruitment and screening took place from August 2016 until August 2017 by the physicians from the neurological rehabilitation unit at Hospital d’Esperança in Barcelona. Potential participants were recruited and screened among the outpatients that visited the physicians for the yearly control at the hospital. This convenience sampling ensured a representative sample of community-dwelling chronic stroke patients. The inclusion criteria were as follows: a) cognitive impairment due to a first-ever stroke (Montreal Cognitive Assessment [60], MoCA < 26), b) no severe upper limb motor disability (Medical Research Council Scale for stroke assessment [61], MRC > 2), c) age between 45 and 75 years old and d) chronic state (more than 6 months after stroke but less than 10 years). The exclusion criteria were as follows: a) severe cognitive incapacity that prohibits the execution of the experiment, b) severe impairments like spasticity, communication disabilities (aphasia or apraxia) and perceptual or physical impairments that would interfere with the correct execution or understanding of the experiment, c) history of severe mental health problems that were present in the acute or subacute phase and d) presence of hemianopia. The reason for including patients with first-ever stroke only is that current literature is inconclusive whether a recurrent stroke enhances existing cognitive deficits or not [62, 63]. Inclusion and exclusion criteria, as well as stroke etiology, were checked by the physicians using standard clinical tools, the clinical history of the patient, and clinical appraisal. As there is no existing study from which estimations for our primary outcome measurements could have been obtained, the sample size had to be predicted instead of calculated through a power analysis. Based on our previous experiments that proved to be achievable with the resources and time available [32, 64], other trials with similar interventions [38, 65] and the doctor’s estimation of recruitment pace, a sample size of 30 participants was deemed adequate. The trial concluded when the sample size for a complete case analysis was reached. The Consolidated Standards of Reporting Trials (CONSORT) statement was used to report the trial.

**Fig. 1:**
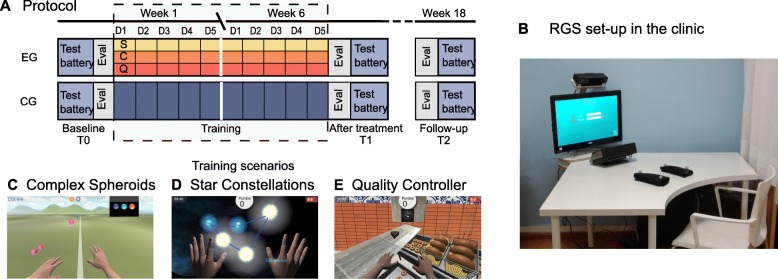
Experimental protocol and set-up. **a** The protocol lasted 18 weeks in total, 6 weeks of training, and 3-months follow-up period. **b** The set-up of the EG in the hospital consisted of a desktop computer, a Microsoft Kinect and two wristbands with reflective markers that are worn by the patient. A Tobii EyeTracker T120 tracked the eye movement of the patient during the training. The Kinect detects the reflective markers and transposes the movement of the patient's real arms onto the virtual arms of the avatar in the training scenarios. The patients are seated at a table, and the three training scenarios (**c** Complex Spheroids, **d** Star Constellations, and **e** Quality Controller) are shown on the screen always in the same order. Besides the automated adaptive difficulty mechanism and the embodied training, the system incorporates further principles of neurorehabilitation including the provision of multisensory feedback, feedback of results, variable and structured practice as well as promoting the use of the paretic limb. *C* Star Constellations, *CG* control group, *D* day, *EG* experimental group, *Eval* VR evaluation, *Q* Quality Controller, *RGS* Rehabilitation Gaming System, *S* Complex Spheroids

Eligible patients that gave their written consent to participate were assessed by a neuropsychologist using the following tests: a neuropsychological test battery, additional clinical outcome scales and two VR assessments — at baseline (T0), after the intervention (T1) and at 3 months follow-up (T2). All assessments were conducted in the aforementioned order in one session, in the median 4 days before and 3 days after the intervention period. At baseline, the patients were randomized by the experimenter either into an experimental group (EG) or a control group (CG) using a custom-made computerized minimization procedure based on the open-source software OxMAR [66] to ensure balanced groups across the baseline characteristics (gender, age, days after stroke, MoCA, Mini-Mental State Examination (MMSE), Barthel Index (BI) and Fugl-Meyer Assessment for the upper extremity (FM-UE)) and all the scores of the neuropsychological test battery (see section Outcome Measurements). Specifically, the measurements were stratified (dummy-coded) as follows: For the neuropsychological test battery and as well as the MoCA, MMSE, BI and FM-UE established cut-offs for the categories “no impairment”, “mild impairment”, “moderate impairment” or “severe impairment” were taken from normative data (see Additional file 1: Statistical Procedure), for age, the cut-off was set at 65, for days after stroke at 590 days and gender was categorized in male and female. First, a new patient would be stratified (dummy-coded with 0’s and 1’s) according to these cut-offs. Then the sums of the strati between the groups with the new patient added are compared. The patient is then either allocated to the group with the lower sum or if the sums are equal randomly allocated with a 50% chance for either group. The first four recruited patients were assigned using a computer-generated list of random numbers only known to the experimenter. Due to the nature of the intervention and personal resources, participants and the experimenter were not blind to the group allocation. The neuropsychologist was not informed about group allocation. However, since the assessments and the intervention took place in the same hospital it could not be prevented that some patients would cross path with the assessor. All patients underwent a six week long, daily training of 30 min, five times per week (Fig. 1a).

### Experimental intervention

The EG played each day three cognitive training scenarios of 10 min each. The dose of training was estimated to be adequate based on the results from our previous studies in the motor domain [32] and the currently reported average intervention time [12]. The training was provided through the RGS set-up (Fig. 1b), a VR-based rehabilitation tool. After an initial introduction and explanation of the scenarios on the first day, the patients interacted independently with RGS. Every day the therapist on duty would place the patient in front of the motion capture sensor and the screen, log in to the system, and commence the intervention. Only a few patients required help with putting on and taking off the markers and to change between the training protocols. Apart from this, the therapist did neither assist during the intervention, check adherence to the goal of the task, nor provide any feedback to the patient. The therapist was, however, allowed to help when technical issues or computer problems arose. The data generated through the interaction with the system was automatically stored in a remote secured database at the experimenter’s institution. RGS has been validated in several clinical studies to be effective in functional motor recovery [32, 44, 64, 67]. With this pilot study, we extended the RGS framework of embodied training, where the patient controls a virtual avatar on a computer screen, with conjunctive cognitive training scenarios which we call ACCT. Besides, RGS incorporates an automated mechanism that adapts the difficulty of the training to each patient’s ability [59]. It is thought that training efficiency and engagement is maximal if the challenge level is optimal regarding performance, perceived difficulty and fatigue, e.g., a person learns maximally if the experienced difficulty is neither too low nor too high [68]. To maintain the perceived challenge within a scenario at a consistent level, the algorithm used here adapts task parameters (for instance the speed of moving objects, or the number of items that need to be memorized) which influence the actual difficulty based on the patient’s ongoing performance in the task [59]. Thus, when the patient is reaching a high level of performance, the algorithm makes the task more difficult, while when the patient’s performance drops, the algorithm makes the task easier. The task parameters were selected to train skills that underlie the cognitive domains investigated here, and based on existing literature on recommendations for effective cognitive rehabilitation. The skills and task parameters were combined into two training scenarios in order to provide the patients with multidomain exercises. The training scenarios and their task parameters are explained hereinafter. Performance is calculated as the relative success rate, e.g. the number of successful attempts over a given number of trials and the algorithm’s objective is to maintain it around 70–80%. In this study, we assume that the levels of the task parameters reflect the individual cognitive impairment levels. The tasks are briefly explained in the following paragraphs. Detailed information can be found in Additional file 1: Experimental Intervention and Additional file 2: Movie S1.

The Complex Spheroids scenario aims at training basic attention and memory ability without an automated adaptation of difficulty (Fig. 1c). It requires the patient to intercept approaching colored spheres by following a predefined sequence indicated at the top right corner of the screen.

In the Star Constellations scenario (Fig. 1d), a visuospatial short-term memory task [37], the patients must remember a given subset of stars in a constellation and reproduce them after a delay period. The difficulty level of four task parameters is adapted in this scenario: 1) The complexity and spatial extension of the constellations (seven levels) aim to train spatial attention and spatial memory. This parameter addresses the recommendation to offer a unique sequence of stimuli in each trial during working memory training [37], to progress from simpler to more complex tasks in executive function training [10] and to train the ability to detect and deploy attention to all sides of space [12]. 2) and 3) The number of stars in a subset and the time interval between their appearance should aid the training of working memory [37]. 4) The length of the delay period progressively challenges memory delayed recall. This parameter aids the training of internal strategies (visual imagery) which are recommended for memory training [6]. The countdown of the delay period serves as a non-spatial alerting intervention to train sustained attention [19].

In the Quality Controller scenario (Fig. 1e), patients are presented with two tasks concurrently. In the right workspace, doughnuts must be taken out of a fryer when their cooking time ends. In the left workspace, the patient must detect defective candies on a conveyor belt. The difficulty level of five task parameters is adapted in this scenario: 1) and 2) The speed of the conveyor belt, and the interval between appearing candies aim to train alertness. These parameters address speed-of-processing training that fosters visual search skills to identify and locate visual information quickly and in a divided-attention format [69]. 3) The ratio between defective and good candies is thought to promote selective and sustained attention. This parameter addresses the ability to focus on specific stimuli while ignoring irrelevant ones in attention training [12]. 4) The baking time of the doughnuts should train the ability to inhibit prepotent responses in executive function training [10]. 5) The time given to take the doughnuts out of the fryer should aid the training of initiation of behaviours in executive function training [10]. The alarm clock that signals when the doughnuts are ready should foster readiness to respond and, therefore, alertness and arousal [12]. The patient has to take care of the two spatially distributed tasks simultaneously; therefore, training divided attention ability, which is essential for multitasking and spatial attention [12]. The scenario should address bottom-up stimulus-driven alerting in spatial neglect [51] by promoting visual search, which improves voluntary exploration of the contralesional space [29]. It further addresses problem-solving and strategy formation techniques required in executive function [6, 10].

### Control intervention

The CG received from the experimenter at the hospital a folder with 30 individual cognitive tasks that had to be completed at home (e.g., crosswords, spot the ten differences, draw complex figures reversed, or complete sentences) during 30 min at each workday. The tasks were selected by the neuropsychologist to overlap with the cognitive abilities essential in the experimental tasks (spatial awareness, attention, memory, executive ability) and to be representative of what would be generally suggested to community-dwelling patients for at-home training. The adherence to the control intervention was not monitored during the experiment. The patients were asked to write down the date and the time spent on each task and return the folder after 6 weeks. After the treatment, the patient would return the folder to the experimenter, who checked that the exercises were completed and asked the patients whether they had any difficulties fulfilling their task.

### Outcome measures

The primary outcome measurements were four averaged standardized composite scores (ASCS) for attention, memory, executive function, and spatial awareness calculated from the neuropsychological test battery. The neuropsychological test battery was compiled by the neuropsychologist and covered the four cognitive domains. For attention, we chose the Corsi Block Tapping Test Forward (Corsi F) [70], the Trail Making Test A (TMT A) [71], and the Wechsler Adult Intelligence Scale-Fourth Edition (WAIS) [72] Digit Span Forward (WAIS F). For memory, we selected the Corsi Block Tapping Test Backward (Corsi B) [70], the Rey Auditory Verbal Learning Test Immediate (RAVLT I) and Delayed Recall (RAVLT D) [73], and the WAIS Digit Span Backward (WAIS B). Executive function was covered by the TMT B, the WAIS Digit Symbol Coding (WAIS C) and the Frontal Assessment Battery (FAB) [74], and lastly, spatial awareness consisted of the Star Cancellation Test (Star) [75]. The standard scoring and Spanish test versions were used. Secondary outcomes were clinical scales that allowed us to check for additional effects of the treatment and consisted of the MoCA [60], the BI [76], the FM-UE [77], the Hamilton Depression Rating Scale (HAM-D) [78] and the MMSE [79]. The HAM-D outcome is only available for 21 subjects, as it was added after the first analysis of data [80]. Although patients with a history of severe mental health problems should have been excluded by our exclusion criteria, we suspected that mood might influence the results. In addition, the protocol included two VR assessments that will be analyzed in separate reports.

### Statistical procedure

Since normality testing (Lilliefors test of normality) pointed out that most of our data except HAM-D were not normally distributed, we used non-parametric testing. Baseline characteristics and outcome measures were compared between groups using Wilcoxon’s rank-sum test (*Ws*) for interval and ordinal variables, and Pearson’s chi-square test (*χ*2) for nominal variables. Spearman’s correlation was used to assess how well the task parameters of each training scenario (median after 1 week of training) correlated with the neuropsychological test battery at baseline. For the primary outcomes, the individual test scores for each cognitive assessment were converted into standardized z-scores, using the mean and standard deviation (SD) of normative age-adjusted data. By averaging the z-scores, the ASCS for each domain were obtained. To obtain a measurement of generalized cognitive functioning, we took the median of the patient’s ASCS within each domain. Each patient’s ASCS per domain was stratified according to its SD from the normative mean to obtain the impairment level in each domain. The correlation within ASCS was evaluated using Spearman’s correlation. First, a within-group analysis was performed, evaluating the changes of ASCS scores and secondary outcomes over time across the three assessment points of the study (baseline T0, after treatment T1 and follow-up T2) using the Friedman’s ANOVA test statistic (${\chi}_F^2$). Then a post hoc analysis was performed using Wilcoxon’s sign rank test (*T*) comparing the scores after treatment and at follow-up with baseline. For the between-group analysis, the improvement after treatment (T1 – T0) and at follow-up (T2 – T0) was compared between EG and CG using the Wilcoxon’s rank-sum test (*Ws*). A complete case analysis and a last observation carried forward analysis were performed to deal with missing data. Significant results were only accepted when confirmed by both analyses. For the depression subgroup analysis, the improvement in ASCS was evaluated with a linear regression, in addition to the within- and between-group analysis. A detailed description of the statistical procedure can be found in the supplementary material (see Additional file 1: Statistical Procedure).

## Results

**Fig. 2:**
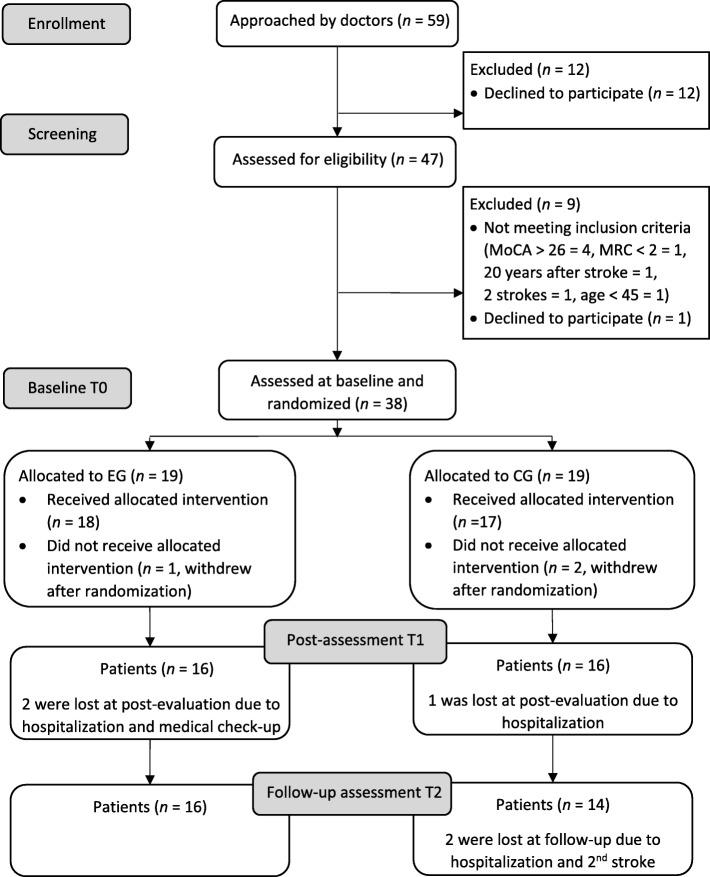
CONSORT flow diagram. *CG* control group, *EG* experimental group, *MoCA* Montreal Cognitive Assessment, *MRC* Medical Research Council Scale for stroke assessment

We approached 59 chronic stroke patients, of which 47 agreed to participate and were assessed for eligibility (CONSORT flow diagram Fig. 2). Thirty-eight eligible individuals were assessed at baseline and randomized into EG (*n* = 19) and CG (*n* = 19). Their baseline characteristics can be found in Table 1. There were no differences between the groups in their baseline characteristics or in any of their baseline primary or secondary outcome measures. Three patients (CG = 2, EG = 1) withdrew after randomization. Thirty-five patients (EG = 18, CG = 17) completed the 6 weeks intervention program. In the CG, one patient was lost at post-assessment and two at follow-up. In EG, two patients were lost at post-assessment, resulting in 30 valid cases (EG = 16, CG = 14). Except for one patient that was able to complete only nine tasks, all the patients in the CG did complete all the paper and pencil tasks. However, only two patients noted down the time they spent on each task. Based on their reports, they spent between 20 to 30 min on each task, except for a few tasks they were able to finish in 5 minutes, and that should be replaced in the larger trial.

**Table 1:** Patient characteristics and secondary outcome measurements at baseline

Characteristics	EG (*n* = 19)	CG (*n* = 19)	*p*
	n (%)		*χ*^2^
Gender, female	8 (42.11%)	7 (36.84%)	.33
Impaired limb, right	8 (42.11%)	5 (26.32%)	.62
Etiology			.39
Ischemic	10 (52.63%)	14 (73.68%)	
Hemorrhagic	7 (36.84 %)	5 (26.32%)	
Capsulo lenticular	1 (5.26%)	--	
Undefined	1 (5.26%)	--	
	Mean (SD) – Median [2.5^th^ – 97.5^th^ percentile]		*W*_*s*_
Age, years	63.63 (6.73) – 63 [53.00 – 76.00]	67.21 (6.45) – 68 [57.00 – 76.00]	.15
Days after stroke	851.16 (805.26) – 620 [192.00 – 3211.00]	12625.9 (1376.1) – 625 [190.00 – 5805.00]	.32
MoCA	20.32 (3.92) – 21 [12.00 – 25.00]	20.05 (3.79) – 20 [12.00 – 25.00]	.76
MMSE	27 (2.08) – 27 [23.00 – 30.00]	26.68 (2.31) – 27 [22.00 – 29.00]	.79
MRC	3.79 (0.71) – 4 [3.00 – 4.00]	3.26 (1.28) – 4 [3.00 – 4.00]	.36
FM-UE	53.79 (14.36) – 60 [15.00 – 66.00]	50.44 (19.45) – 62 [5.00 – 66.00]	.74
BI	95 (7.63) – 100 [80.00 – 100.00]	86.11 (20.04) – 95 [20.00 – 100.00]	.15

*BI* Barthel Index, *CG* control group, *EG* experimental group, *FM-UE* Fugl-Meyer Assessment for the upper limb, *SD* standard deviation, *MoCA* Montreal Cognitive Assessment, *MRC* Medical Research Council Scale, *MMSE* Mini-Mental State Examination, *X*^*2*^ Pearson Chi-square statistic, *W*_*s*_ Wilcoxon’s rank-sum test

**Fig. 3:**
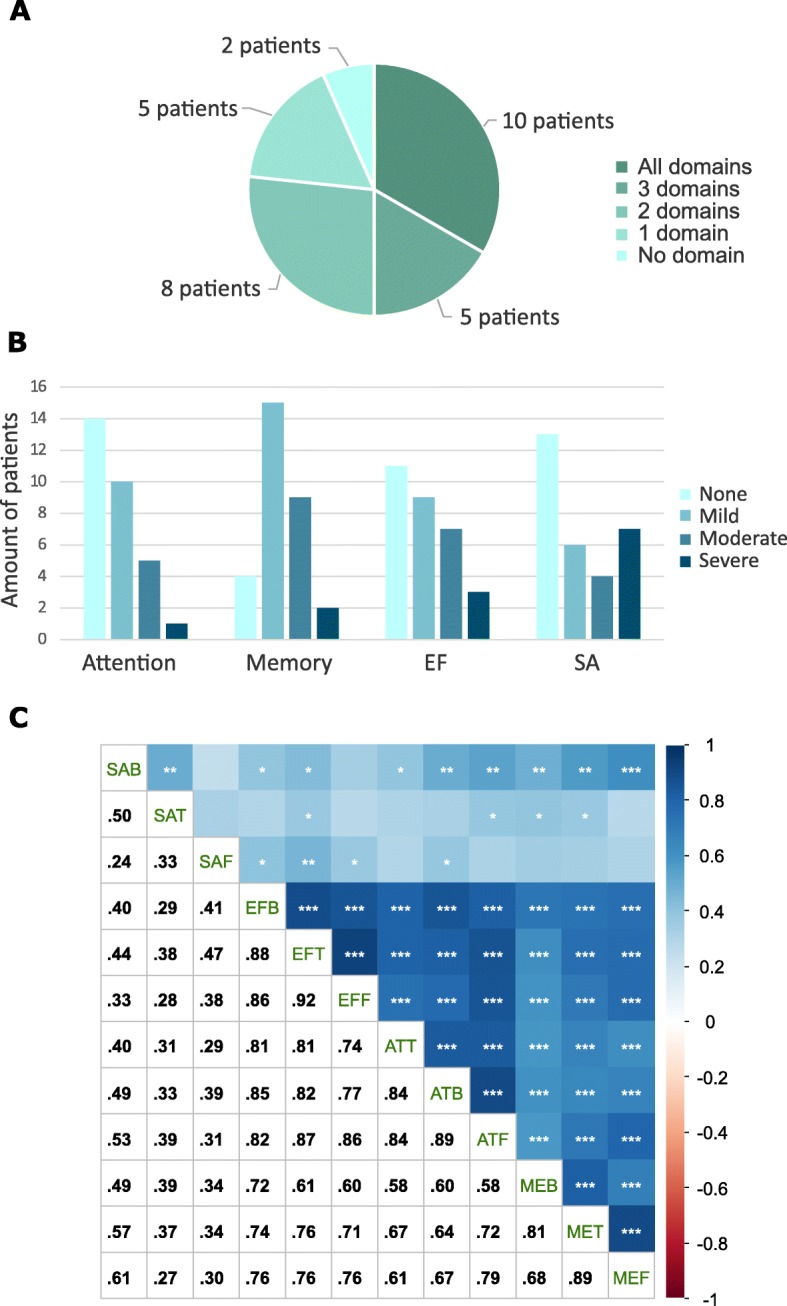
Impairment distribution and correlation at baseline. **a** Distribution of the number of domains impaired. **b** Distribution of severity per domain. **c** The ASCS for attention, memory, and executive function positively correlate at baseline, after treatment and follow-up. ASCS for spatial awareness seems to correlate weakly at baseline with the other domains, but this correlation is not visible after treatment and at follow-up. Significant *p*-values are indicated as * *p*-value < .05, ** *p*-value < .01, *** *p*-value < .001 and the color scale represents the correlation coefficient (Spearman’s r). *ASCS* averaged standardized composite score, *ATB* attention ASCS at baseline, *ATF* attention ASCS at follow-up, *ATT* attention ASCS after treatment, *EF* executive function, *EFB* executive function ASCS at baseline, *EFF* executive function ASCS at follow-up, *EFT* executive function ASCS after treatment, *MEB* memory ASCS at baseline, *MEF* memory ASCS at follow-up, *MET* memory ASCS after treatment, *SA* spatial awareness, *SAB* spatial awareness ASCS at baseline, *SAF* spatial awareness after follow-up, *SAT* spatial awareness at follow-up

Most of the patients showed an impairment in all four domains at baseline (Fig. 3a). Only five patients showed an impairment in a single domain, whereas two patients were better as the normative mean in all domains. Every domain contains a spread across all impairment levels (Fig. 3b). The Spearman’s correlation revealed that the ASCS of attention, memory and executive function, but not spatial awareness, of all patients together correlated significantly at baseline, after treatment, and at follow-up (Fig. 3c).

**Fig. 4:**
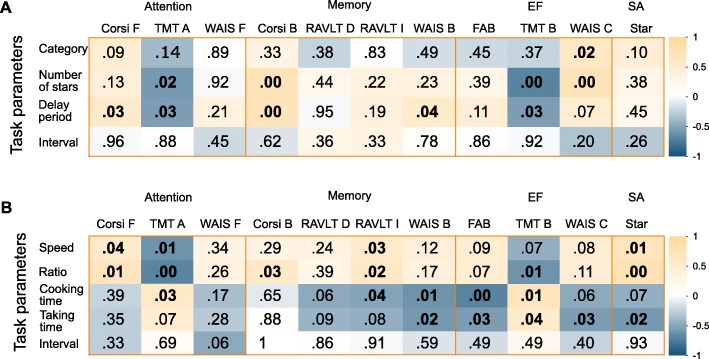
Correlations between the task parameters of the training scenarios and the neuropsychological test battery. **a** The task parameters of the Star Constellations scenario are the constellation complexity level (category), the number of stars in the subset (number of stars), the time interval between their appearance (interval) and the length of the delay period (delay period). **b** The task parameters of the Quality Controller scenario are the speed of the conveyor belt (speed), the time interval between the appearance of the candies (interval), the ratio between defective and good candies (ratio), the baking time of the doughnuts (baking time) and the time given to take the doughnuts out of the fryer (taking time). The number represents the *p*-value and the color scale represents the correlation coefficient (Spearman’s r). *Corsi B* Corsi Block Tapping Test Backward, *Corsi F* Corsi Block Tapping Test Forward, *EF* executive function domain, *FAB* Frontal Assessment Battery, *RAVLT* Rey Auditory Verbal Learning Test, *RAVLT I* RAVLT Immediate, *RAVLT D* Delayed Recall, *Star* Star Cancellation Test, *SA* spatial awareness domain, *TMT A* Trail Making Test A, *TMT B* Trail Making Test B, *WAIS* Wechsler Adult Intelligence Scale-Fourth Edition, *WAIS B* WAIS Backward, *WAIS C* WAIS Digit Symbol Coding, *WAIS F* WAIS Digit Span Forward

In Fig. 4, we show the correlations between the median task parameters of each scenario, which regulate the difficulty of the training, after the first week of intervention, and the neuropsychological test battery at baseline in EG (*n* = 16). The analysis revealed that in the Star Constellations scenario (Fig. 4a) the median number of stars that a patient was able to remember correlated well with the scores in TMT A (*r*_*s*_ = −.57, *p* < .05), Corsi B (*r*_*s*_ = .67, *p* < .01), TMT B (*r*_*s*_ = −.69, *p* < .01) and WAIS C (*r*_*s*_ = .69, *p* < .01). Similarly, the median delay period achieved correlated well with the scores in TMT A (*r*_*s*_ = −.56, *p* < .05) and Corsi B (*r*_*s*_ = .68, *p* < .01), and moderately with WAIS C (*r*_*s*_ = .46, *p* = .07). In addition, it correlated with Corsi F (*r*_*s*_ = .54, *p* < .05) and WAIS B (*r*_*s*_ = −.56, *p* < .05). Moreover, there was a correlation between the median constellation complexity level and WAIS C (*r*_*s*_ = .59, *p* < .05). For the Quality Controller scenario (Fig. 4b) several correlations between task parameters and neuropsychological test battery scores have been found as well. The median speed of the conveyor belt and the ratio between good and defective candy correlated well with Corsi F (*r*_*s*_ = .53, *p* < .05 and *r*_*s*_ = .65, *p* < .01), TMT A (*r*_*s*_ = −.61, *p* < .05 and *r*_*s*_ = −.69, *p* < .01), RAVLT I (*r*_*s*_ = .53, *p* < .05 and *r*_*s*_ = .57, *p* < .05), TMT B (*r*_*s*_ = −.46, *p* = .07 and *r*_*s*_ = −.62, *p* < .05) and Star (*r*_*s*_ = .65, *p* < .01 and *r*_*s*_ = .75, *p* < .001). On the other hand, the median baking time and the median time to take out the doughnuts correlated with TMT A (*r*_*s*_ = .54, *p* < .05 and *r*_*s*_ = .46, *p* = .07), RAVLT I (*r*_*s*_ = −.53, *p* < .05 and *r*_*s*_ = −.45, *p* = .08), WAIS B (*r*_*s*_ = −.60, *p* < .05 and *r*_*s*_ = −.58, *p* < .05), FAB (*r*_*s*_ = −.70, *p* < .01 and *r*_*s*_ = −.53, *p* < .05), TMT B (*r*_*s*_ = .65, *p* < .01 and *r*_*s*_ = .53, *p* < .05), WAIS C (*r*_*s*_ = −.47, *p* = .06, and *r*_*s*_ = −.53, *p* < .05) and Star (*r*_*s*_ = −.47, *p* = .07 and *r*_*s*_ = −.58, *p* < .05).

**Fig. 5:**
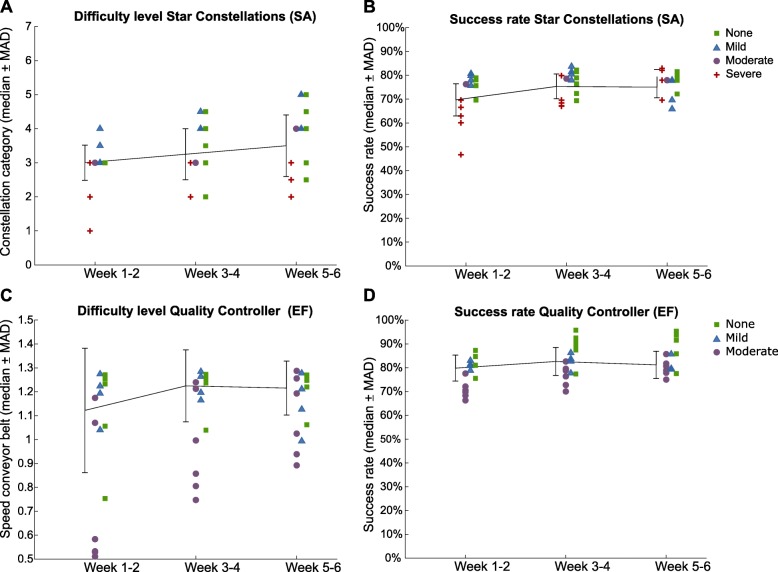
The relationship between impairment level, difficulty achievement, and performance (success rate) within training scenarios. **a** Difficulty achievement in the Star Constellations scenario and **c** in the Quality Controller scenario. The task parameter in Star Constellations is the constellation complexity level, and in Quality Controller the speed of the conveyor belt. Success rate (number of successful attempts over all possible trials in percentage) for Star Constellations (**b**) and Quality Controller (**d**). Possible trials in Star Constellations are the total number of constellations shown in a session. In Quality Controller success rate represents the true positives of all defective candies in a session. Solid line and error bars represent median and median absolute deviation per 10 sessions (two weeks), data points represent individual patients stratified according to their impairment level in spatial awareness domain for Star Constellations and executive function domain for Quality Controller at baseline: severe (red cross), moderate (violet circle), mild (blue triangle) and no impairment (green square). *EF* executive function domain, *MAD* median absolute deviation, *SA* spatial awareness domain

The algorithm adapted the task parameters well to the individual impairment level in EG (*n* = 16), ensuring a stable success rate while training (Fig. 5). For instance, in the Star Constellations scenario, stratifying patients according to their impairment level in the spatial awareness domain at baseline revealed that more severe patients achieved lower difficulty levels than less impaired ones (Fig. 5a). Throughout the training, however, the achieved difficulty level seemed to increase across all severity levels. Although the task parameter levels differed for each patient, the success rate remained stable at around 70% (Fig. 5b). The same pattern can be observed in the Quality Controller scenario (Fig. 5c and d). Here, however, the achieved task parameter might not have been challenging enough for non-impaired patients as their performance was around 90% (Fig. 5d).

**Fig. 6:**
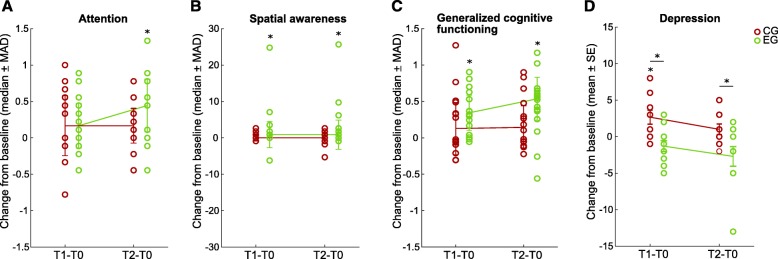
Main findings in ASCS scores and subgroup analysis. Change in (**a**) attention ASCS, (**b**) spatial awareness ASCS, (**c**) generalized cognitive functioning ASCS, and (**d**) depression (HAM-D) from baseline to after treatment (T1-T0) and to follow-up (T2-T0) for the experimental group (EG, green) and control group (CG, red). The individual data for each subject is indicated with dots. Negative numbers in HAM-D mean improvement (less depression). The ASCS scores change for memory, and executive function can be found in Additional file 1: Figure S1. *MAD* median absolute deviation, *SE* standard error of the mean

In Table 2, we show the descriptive data of the ASCS for every domain at baseline (T0), after treatment (T1) and at follow-up (T2) as well as the *p*-values of the within-group analysis for the complete cases (EG = 16, CG = 14). The data for the last observation carried forward analysis (EG = 19, CG = 19) can be found in the Additional file 1: Table S1. We found a significant change in ASCS over time for the EG in the attention domain (${\chi}_F^2$ (2) = 9.57, *p* < .01), in the spatial awareness domain (${\chi}_F^2$ (2) = 11.23, *p* < .01) and in the generalized cognitive functioning (${\chi}_F^2$ (2) = 14.00, *p* < .001) in the complete case analysis (Fig. 6a-c), which was confirmed by the last observation carried forward analysis. In the attention domain, the post hoc analysis revealed significantly higher scores at T2 (*T* = 84.5, *r* = .48, *p* < .01) as compared to baseline. In the spatial awareness domain, the post hoc analysis revealed significant higher scores at T1 (*T* = 47, *r* = .35, *p* < .05) and at T2 (*T* = 63, *r* = .47, *p* < .01) as compared to baseline. In the generalized cognitive functioning, the post hoc analysis indicated significant higher scores at T1 (*T* = 130, *r* = .59, *p* < .01) and at T2 (*T* = 123, *r* = .52, *p* < .01) as compared to baseline. For the CG, no significant change over time was found, although the memory domain yielded significantly higher scores at T1 (*T* = 86, *r* = .56, *p* < .05) that was confirmed by the last observation carried forward analysis. No significant results for either group were found in the executive function domain. Neither we found significant differences between the groups in the complete case analysis that would have been confirmed in the last observation carried forward analysis (Table 3). The descriptive statistics for every test in the neuropsychological test battery can be found in Additional file 1: Table S2.

**Table 2:** ASCS at baseline (T0), after treatment (T1) and follow-up (T2) and the *p*-values for the within-group analysis of the change over time for the complete case analysis

ASCS	EG (*n* = 16)				CG (*n* = 14)			
	Mean (SD) – Median [2.5^th^ – 97.5^th^ percentile]			*p*	Mean (SD) – Median [2.5^th^ – 97.5^th^ percentile]			*p*
	T0	T1	T2	${\chi}_F^2(2)$	T0	T1	T2	${\chi}_F^2(2)$
Attention	-0.35 (0.88) – -0.28 [-2.11 – 1.22]	-0.13 (0.94) – -0.17 [-1.67 – 1.33]	**0.06 (0.92) –** 0.17 [-1.44 – 1.67]**	**.01**	-0.16 (0.83) – 0.11 [-1.78 – 0.89]	0.02 (0.80) – 0.28 [-1.78 – 1.00]	0.03 (0.92) – 0.22 [-1.67 – 1.67]	.25
Memory	-0.76 (0.69) – -0.57 [-2.27 – 0.05]	-0.54 (0.91) – -0.31 [-2.17 – 0.76]	-0.43 (0.91) – -0.30 [-2.19 – 0.89]	.30	-0.72 (0.82) – -0.54 [-2.38 – 0.40]	**-0.52 (0.73) –** -0.44 [-1.78 – 0.56]*	-0.37 (0.83) – -0.52 [-1.37 – 1.52]	.42
EF	-0.34 (1.01) – -0.34 [-1.64 – 1.32]	-0.29 (1.18) – -0.38 [-2.09 – 2.02]	-0.15 (1.19) – 0.15 [-1.97 – 1.68]	.43	-0.45 (1.38) – -0.27 [-2.67 – 1.79]	-0.28 (1.33) – -0.02 [-2.60 – 2.02]	-0.28 (1.40) – -0.21 [-2.60 – 1.91]	.47
SA	-2.88 (6.57) – -0.39 [-25.17 – 0.50]	**-0.67 (3.95) –** 0.50 [-15.43 – 0.50]*	**0.33 (0.36) –** 0.50 [-0.39 – 0.50]*	**.00**	-0.58 (1.44) – 0.05 [-3.93 – 0.50]	-0.20 (1.44) – 0.50 [-4.81 – 50]	-0.52 (1.90) – 0.50 [-6.68 – 0.50]	.53
GCF	-0.56 (0.79) – -0.44 [-1.92 – 0.39]	**-0.20 (0.80) –** -0.10 [-1.64 – 0.91]**	**-0.12 (0.83) –** 0.25 [-1.56 – 0.99]**	**.00**	-0.38 (0.90) – 0.00 [-2.00 – 0.69]	-0.17 (0.81) – 0.10 [-1.93 – 0.75]	-0.16 (0.94) – 0.06 [-1.98 – 1.59]	.93

The change over time within each group was evaluated using Friedman’s ANOVA test statistic ${\chi}_F^2$ (degrees of freedom). The table shows the *p*-values (*p*) with values below .05 highlighted in bold. For the post hoc analysis the Wilcoxon’s sign rank test *T* was used, and significant comparisons with respect to baseline are indicated with * for *p*-values < .05 and ** for *p*-values < .01. *ASCS* average standardized composite score, *CG* control group, *GCF* generalized cognitive functioning, *EF* executive functioning, *EG* experimental group, *SA* spatial awareness

**Table 3:** Between-group analysis of baseline ASCS (T0) as well as improvement in ASCS after treatment (T1 – T0) and at follow-up (T2 – T0)

ASCS	EG (*n* = 16)	CG (*n* = 14)	*p*
	Mean (SD) – Median [2.5^th^ – 97.5^th^ percentile]		*W*_*s*_
Attention			
T0	-0.35 (0.88) – -0.28 [-2.11 – 1.22]	-0.16 (0.83) – 0.11 [-1.78 – 0.89]	.39
T1 – T0	0.22 (0.39) – 0.17 [-0.44 – 0.89]	0.17 (0.50) – 0.17 [-0.78 – 1.00]	.80
T2 – T0	0.41 (0.46) – 0.44 [-0.44 – 1.33]	0.19 (0.32) – 0.17 [-0.44 – 0.78]	.21
Memory			
T0	-0.76 (0.69) – -0.57 [-2.27 – 0.05]	-0.72 (0.82) – -0.54 [-2.38 – 0.40]	.85
T1 – T0	0.21 (0.50) – 0.23 [-0.70 – 1.22]	0.20 (0.31) – 0.10 [-0.30 – 0.76]	.82
T2 – T0	0.33 (0.65) – 0.47 [-0.73 – 1.71]	0.34 (0.51) – 0.34 [-0.47 – 1.29]	.79
EF			
T0	-0.34 (1.01) – -0.34 [-1.64 – 1.32]	-0.45 (1.38) – -0.27 [-2.67 – 1.79]	.92
T1 – T0	0.05 (0.61) – 0.04 [-1.55 – 0.96]	0.17 (0.54) – 0.17 [-0.30 – 0.76]	.57
T2 – T0	0.19 (0.62) – 0.22 [-0.85 – 1.55]	0.17 (0.56) – 0.18 [-1.25 – 1.00]	.79
SA			
T0	-2.88 (6.57) – -0.39 [-25.17 – 0.50]	-0.58 (1.44) – 0.05 [-3.93 – 0.50]	.45
T1 – T0	2.21 (6.55) – 0.88 [-6.19 – 24.78]	0.38 (1.08) – 0.00 [-0.88 – 2.65]	.24
T2 – T0	3.21 (6.57) – 0.88 [-0.88 – 25.66]	0.06 (1.95) – 0.00 [-5.31 – 2.65]	.10
GCF			
T0	-0.56 (0.79) – -0.44 [-1.92 – 0.39]	-0.38 (0.90) – 0.00 [-2.00 – 0.69]	.49
T1 – T0	0.36 (0.36) – 0.34 [-0.06 – 0.90]	0.21 (0.45) – 0.13 [-0.31 – 1.27]	.12
T2 – T0	0.44 (0.42) – 0.54 [-0.56 – 1.17]	0.22 (0.37) – 0.14 [-0.22 – 0.90]	.12

The differences at baseline and in improvement from baseline at T1 and T2 between groups were evaluated using the Wilcoxon rank-sum test *W*_*s*_. The table shows the *p*-values (*p*) of the comparisons. *ASCS* average standardized composite score, *CG* control group, *GCF* generalized cognitive functioning, *EF* executive functioning, *EG* experimental group, *LOCF* last observation carried forward, *SA* spatial awareness

Additional file 1: Table S3 shows the results for the secondary outcomes. We found a significant change over time only in MMSE for CG (${\chi}_F^2$ (2) = 7.14, *p* < .05). Post hoc analysis revealed a significant difference between T0 and T2 (*T* = 62.5, *r* = .72, *p* < .01). For the EG we found that FM-UE after treatment was significant different from baseline (*T* = 43, *r* = .61, *p* < .05) and that this improvement was also significant different from the improvement of the CG (*Ws* = 288.5, *z* = 2.22, *r* = .40, *p* < .05). No other significant results in the secondary outcomes were found (see Additional file 1: Figure S2).

In Table 4, we report the results of the within-group analysis for the depression subgroup analysis (EGD = 11, CGD = 10). The CGD shows a significant worsening in the HAM-D at T1 (*T* = 45, *r* = .72, *p* < .01) as compared to baseline. At T1, the depression level of the CGD was significantly higher in comparison to the EGD (*Ws* = 81.5, *z* = − 2.76, *r* = − .60, *p* < .01) and this difference remained significant at T2 (*Ws* = 92, z = − 2.03, *r* = − .44, *p* < .05), see Table 5 and Fig. 6d. We observed a significant effect of time for EGD in the attention domain (${\chi}_F^2$ (2) = 10.82, *p* < .01) and in the generalized cognitive functioning domain (${\chi}_F^2$ (2) = 9.8, *p* < .01). Post hoc analysis in the attention domain revealed a significant difference between T0 and T2 (*T* = 43.5, *r* = *0*.53, *p* < .05) and between T1 and T2 (*T* = 40.5, *r* = .46, *p* < .05). Post hoc analysis in the generalized cognitive functioning domain showed a significant difference from T0 to T1 (*T* = 53, *r* = .57, *p* < .01) and from T0 to T2 (*T* = 53, *r* = .57, *p* < .01). In addition, we found a difference between the two groups improvement at T1 in generalized cognitive functioning (*W*_*s*_ = 151, *r* = .45, *p* < .05). For CGD, no change over time was found. These results are similar to what was found in the analysis of the whole study sample. On the other hand, we could only confirm a reduced influence of the level of depression on the performance on the neuropsychological test battery. Of the eleven tests included in our battery, three correlated with the HAM-D at baseline (Corsi F: *r* = − .69, *p* < .05, TMT A: *r* = .45, *p* < .05, TMT B: *r* = .47, *p* < .05). These correlations disappeared after the treatment and at follow-up.

**Table 4:** Depression subgroup analysis. HAM-D and ASCS at baseline (T0), after treatment (T1) and follow-up (T2) and the *p*-values for the within-group analysis of the change over time

Measures	EGD (*n* = 11)				CGD (*n* = 10)			
	Mean (SD) – Median [2.5^th^ – 97.5^th^ percentile]			*p*	Mean (SD) – Median [2.5^th^ – 97.5^th^ percentile]			*p*
	T0	T1	T2	${\chi}_F^2(2)$	T0	T1	T2	${\chi}_F^2(2)$
HAM-D	6.64 (5.46) – 5.00 [0.00 – 15.00]	5.45 (4.89) – 3.00 [0.00 – 13.00]	4.18 (3.34) – 3.00 [0.00 – 12.00]	.22	5.20 (4.78) – 4.00 [0.00 – 13.00]	7.8 (5.98) –7.50 [0.00 – 19.00]*	6.30 (6.20) – 4.00 [0.00 – 18.00]	.06
Attention	-0.27 (0.95) – -0.22 [-2.11 – 1.22]	-0.10 (0.96) – -0.11 [-1.67 – 0.76]*	0.20 (0.89) – 0.11 [-1.33 – 1.67]*	**.00**	0.01 (0.72) – 0.22 [-1.78 – 0.78]	0.03 (0.78) – 0.28 [-1.78 – 0.89]	0.10 (0.76) – 0.22 [-1.67 – 1.11]	.57
Memory	-0.65 (0.57) – -0.44 [-1.72 – 0.05]	-0.35 (0.85) – -0.09 [-1.97 – 0.76]	-0.18 (0.89) – 0.14 [-1.72 – 0.89]	.27	-0.61 (0.62) –-0.54 [-1.78 – 0.78]	-0.46 (0.62) –-0.29 [-1.78 – 0.48]	-0.36 (0.52) – -0.39 [-1.03 – 0.46]	.72
EF	-0.26 (0.88) – -0.32 [-1.64 – 1.13]	-0.34 (1.08) – -0.53 [-2.09 – 1.13]	-0.04 (0.98) – 0.09 [-1.57 – 1.57]	.11	-0.09 (0.67) – -0.11 [-1.13 – 1.13]	-0.14 (0.78) – 0.02 [-2.09 – 0.76]	-0.11 (0.81) – 0.00 [-1.90 – 1.02]	.61
SA	-1.60 (3.34) – -0.39 [-9.24 – 0.50]	-1.03 (4.78) – 0.50 [-15.43 – 0.50]	0.17 (0.45) – 0.50 [-0.39 – 0.50]	.16	-0.21 (1.43) – 0.50 [-3.93 – 0.50]	-0.21 (1.66) – 0.50 [-4.81 – 0.50]	0.05 (0.75) – 0.50 [-1.27 – 0.50]	.95
GCF	-0.41 (0.73) – -0.30 [-1.80 – 0.39]	-0.11 (0.77) – 0.12 [-1.64 – 0.88]**	0.01 (0.73) – 0.24 [-1.16 – 0.99]**	**.01**	-0.11 (0.61) – 0.06 [-1.63 – 0.53]	-0.07 (0.70) – 0.11 [-1.93 – 0.49]	0.00 (0.53) – 0.09 [-1.15 – 0.64]	.58

The change over time within each group was evaluated using Friedman’s ANOVA test statistic ${\chi}_F^2$ (degrees of freedom). The table shows the *p*-values (*p*) with values below .05 highlighted in bold. For the post hoc analysis the Wilcoxon’s sign rank test *T* was used, and significant comparisons with respect to baseline are indicated with * for *p* < .05 and ** for *p* < .01. *ASCS* average standardized composite score, *CGD* control group, *EF* executive functioning, *EGD* experimental group, *GCF* generalized cognitive functioning, *HAM-D* Hamilton Depression Rating Scale, *SA* spatial awareness

**Table 5:** Between-group analysis in depression subgroup of baseline HAM-D and ASCS (T0) as well as improvement in HAM-D and ASCS after treatment (T1 – T0) and at follow-up (T2 – T0)

Measures	EGD (*n* = 11)	CGD (*n* = 10)	*p*
	Mean (SD) – Median [2.5^th^ – 97.5^th^ percentile]		*W*_*s*_
HAM-D			
T0	6.64 (5.46) – 5.00 [0.00 – 15.00]	5.20 (4.78) – 4.00 [0.00 – 13.00]	.72
T1 – T0	-1.18 (2.40) – -1.00 [-5.00 – 3.00]	2.60 (2.84) – 2.50 [-1.00 – 8.00]	**.01**
T2 – T0	-2.45 (4.32) – 0.00 [-13.00 – 2.00]	1.10 (2.18) – 1.00 [-2.00 – 5.00]	**.04**
Attention			
T0	-0.27 (0.95) – -0.22 [-2.11 – 1.22]	0.01 (0.72) – 0.22 [-1.78 – 0.78]	.26
T1 – T0	0.17 (0.30) – 0.11 [-0.44 – 0.78]	0.02 (0.54) – -0.06 [-0.78 – 1.00]	.23
T2 – T0	0.47 (0.46) – 0.44 [-0.11 – 1.33]	0.08 (0.35) – 0.06 [-0.44 – 0.56]	.07
Memory			
T0	-0.65 (0.57) – -0.44 [-1.72 – 0.05]	-0.61 (0.62) – -0.54 [-1.78 – 0.78]	.92
T1 – T0	0.30 (0.53) – 0.37 [-0.54 – 1.22]	0.15 (0.30) – 0.06 [-0.30 – 0.76]	.46
T2 – T0	0.47 (0.69) – 0.58 [-0.73 – 1.71]	0.25 (0.41) – 0.34 [-0.47 – 0.90]	.31
EF			
T0	-0.26 (0.88) – -0.32 [-1.64 – 1.13]	-0.09 (0.67) – -0.11 [-1.13 – 1.13]	.78
T1 – T0	-0.08 (0.59) – -0.03 [-1.55 – 0.85]	-0.04 (0.47) – -0.02 [-0.96 – 0.59]	.92
T2 – T0	0.22 (0.51) – 0.22 [-0.85 – 1.18]	-0.01 (0.53) – 0.00 [-1.25 – 0.70]	.40
SA			
T0	-1.60 (3.34) – -0.39 [-9.24 – 0.50]	-0.21 (1.43) – 0.50 [-3.93 – 0.50]	.29
T1 – T0	0.56 (3.15) – 0.00 [-6.19 – 7.08]	0.00 (0.83) – 0.00 [-0.88 – 1.77]	.27
T2 – T0	1.77 (3.29) – 0.00 [-0.88 – 9.73]	0.27 (1.25) – 0.00 [-1.77 – 2.65]	.35
GCF			
T0	-0.41 (0.73) – -0.30 [-1.80 – 0.39]	-0.11 (0.61) – 0.06 [-1.63 – 0.53]	.31
T1 – T0	0.29 (0.30) – 0.30 [-0.02 – 0.90]	0.04 (0.37) – -0.08 [-0.31 – 0.76]	**.04**
T2 – T0	0.42 (0.38) – 0.42 [-0.26 – 1.17]	0.11 (0.30) – 0.04 [-0.22 – 0.68]	.07

The differences at baseline and in improvement from baseline at T1 and T2 between depression subgroup were evaluated using the Wilcoxon rank-sum test *W*_*s*_. The table shows the *p*-values (*p*) of the comparisons with values below .05 highlighted in bold. *ASCS* average standardized composite score, *CGD* control group in depression subgroup, *EF* executive functioning, *EGD* experimental group in depression subgroup, *GCF* generalized cognitive functioning, *HAM-D* Hamilton Depression Rating Scale, *SA* spatial awareness

One patient in EGD showed a particularly large improvement of 13 points in HAM-D from T0 to T1. To check if this improvement influenced the results found, we performed the subgroup analysis without this patient. After excluding the patient, we observed that the difference between the groups at T2 loses significance as the *p*-value changes from .04 to .07. However, the EGD group continues to express lower depression levels at T2 (mean of 4.40) than the CGD (mean 6.30). The same patient also showed improvements in attention, memory, and spatial awareness. The exclusion of the patient in the analysis of the cognitive domains did not alter the results found, whether in the subgroup analysis nor in the analysis of the whole sample. We, therefore, did not deem this patient as an outlier that had to be excluded from the analysis.

Next, we wanted to see how the improvement in the cognitive domains influenced the improvement in depression level in our subgroup. We included the improvements in ASCS at T1 (T1 – T0) and T2 (T2 – T0) in a linear regression to estimate the respective depression improvement (Table 6). We found a marginally significant prediction power of improvement in attention ASCS (*t*(17) = − 1.99, *p* = .06) and a significant effect of improvement in memory ASCS (*t*(17) = − 2.35, *p* < .05) to predict the patient’s change in HAM-D from baseline to follow-up. These results indicate that improvement in the domains of attention and memory is positively correlated with improvement in depression .

**Table 6:** Results from a linear regression on improvement in depression

	Estimate (standard error)	*p*-value (df)
Attention		
(Intercept)	−0.36 (1.07)	.74 (17)
Coefficient	3.93 (1.98)	**.06 (17)**
Memory		
(Intercept)	−0.31 (0.98)	.75 (17)
Coefficient	3.33 (1.42)	**.03 (17)**

The table provides the estimates (standard error) and the *p*-value (degrees of freedom) with values below .05 highlighted in bold

## Discussion

In this randomized controlled pilot trial, we tested a novel rehabilitation program in VR that trains several cognitive domains in conjunction. Together with a few other clinical trials [38, 65], we are among the first in addressing the multidimensionality of cognitive impairment after stroke, by providing a VR-based cognitive training that adapts its difficulty optimally to the ability of the patient while providing an embodied training with rewarding feedback [81]. Our data set reveals interesting insights when a heterogeneous sample without a specific cognitive deficit is selected. Similar to prospective studies [1, 2], we see that patients show an impairment in more than one domain. The majority was impaired in all four domains. Also, the impairments in the attention, memory, and executive function domain, but not in the spatial awareness domain, are correlated and remain so over time. The rationale behind the training scenarios is that several cognitive skills can be trained together in a multidomain fashion. With the Star Constellations scenario, we intended to address visuospatial working memory and attentional skills. The correlations between the median task parameters achieved after the first week of training and the scores of the neuropsychological test battery at baseline appears to confirm this intention: TMT A, TMT B, and WAIS C are timed and require online visual tracking ability [82, 83], whereas Corsi F, Corsi B, and WAIS B require working memory skills [84, 85], which in the case of Corsi are paired with a visual component [82]. Besides, we found a correlation of the median constellation complexity level with WAIS C, a test that requires fast decoding of number-symbol combinations [82]. With the Quality Controller scenario, we intended to provide a speeded and distributed dual-task training. The correlations of four task parameters with TMT A and TMT B confirms a strong speed-of-processing and attentional switching component [85], whereas the correlation with Star refers to the visual components trained due to the spatially distributed task. The correlations between the timed task components (baking and taking out time) and the tests of the executive function domain supports the training of inhibition and initiation of responses [30] whereas the correlations with Corsi F, RAVLT I and WAIS B point additionally to a memory component inherent to the training [85–87]. We further demonstrated that our system successfully takes the individual impairment level into account and enables the patients to achieve similar success rates despite varying levels of impairment. The difference in the performance achieved between the two scenarios, especially by the non-impaired patients, might be due to a difference in difficulty between subsequent levels; i.e., in the Quality Controller scenario, the next difficulty level was too hard to achieve, so the patients remained on a lower level thus achieving higher success rates. This illustrates the importance of individualizing training through fine-graded difficulty levels to promote learning and rehabilitation but as well highlights the challenges of doing so [88, 89].

Regarding the four cognitive domains assessed, only the EG shows a significant change over time in attention and spatial awareness. We did not see any significant change in the ASCS over time in the CG, who did cognitive pencil and paper exercises at home. In addition, generalized cognitive functioning increased in EG from baseline to follow-up. We are aware that due to the small sample size in this pilot study and the multiple testing, these results could be spurious, and we can therefore not claim any rehabilitation effect. However, the effect found in generalized cognitive functioning seems to be robust because it includes all ASCS and cannot be driven by the improvement in attention alone. Further, a positive change of attention ASCS and generalized cognitive functioning is still present in the depression subgroup analysis. Interestingly the significant changes in EG were confirmed for the follow-up period, as demonstrated by the post hoc analysis at this time point. We could speculate that this delayed effect of training could mean that the patient incorporated what they learned during the training later in their daily activities, similar to what has been observed in cognitive strategy training [90].

Whether the significant changes in attention and spatial awareness ASCS are clinically relevant is difficult to evaluate, as there is no consensus in literature with regards to the clinically important difference (CID) in neuropsychological test batteries. CIDs reported in studies range from 0.5 SD [91] to 1 SD [92] up to 2 SD [93]. Applying a cut-off of 0.5 SD and 1 SD to our sample (see Additional file 1: Table S4 and S5) shows that still more patients in EG improve even above 1 SD from baseline, especially after follow-up. However, future studies should direct their efforts to find ways to standardize neuropsychological testing and establishing CIDs in well-powered clinical studies.

Regarding the secondary outcomes, the CG showed a significant change in the MMSE over time, with post hoc analysis revealing a significant difference between baseline and follow-up (see Additional file 1: Table S3). On the other hand, no change over time was observed in the MoCA for either group. Interestingly, according to MMSE, only one patient would have been classified as having a cognitive impairment at baseline. This finding is in line with literature, where it was observed that the MoCA is more sensitive to cognitive dysfunction than the MMSE [94]. Also, we used at each assessment point a different test variation of the MoCA, so that the patients never repeated the same exercises. The MMSE, however, is only available in one version, and some exercises resemble the cognitive pencil and paper exercises used in the CG, which might have helped them to succeed in this test.

We further found a significant but small group difference in the FM-UE improvement after treatment in favour of the EG (Additional file 1: Table S3). Although the experimental intervention includes a stronger motor component than the control intervention, motor training was not the focus of the study. Therefore, only patients with sufficient active movement and able to overcome gravity (MRC > 2) were included, although the tasks were accomplished by moving the arms only horizontally supported by a table’s surface. Also, the mean change is below the CID [95] and MDC [96], although four patients surpassed the CID threshold of 4.25 (see Additional file 1: Figure S2) at follow-up. However, in a general stroke population, motor and cognitive deficits likely co-occur [15], and cognitive deficits have a negative effect on functional outcome and independence [3]. It has been stressed out that rehabilitation should combine motor and cognitive training [81]. It would, therefore, be interesting to investigate the effect of the proposed training paradigm that already includes a motor component in patients with lower motor functionality. We believe that patients with more severe motor impairments could easily participate in the ACCT program since no movement against gravity is required, and the adaptive difficulty algorithm could ensure that the arrangement of the interactive elements in the training scenarios does not surpass the patient’s active range of motion. Besides, the ACCT program could be complemented with another adaptive mechanism that aids the completion of goal-oriented movements with the paretic arm through a visual manipulation [32].

Lastly, the subgroup analysis revealed that, compared to the EG, the CG expressed higher depression levels after the intervention. The groups remained significantly different at follow-up. We cannot exclude that the non-blinding of group allocation or that the control task that had to be done at home negatively influenced the depression level in the CG. However, we also see a trend for EG to reduce their depression level. This could be due to the alleviation of rumination, a known symptom of depression, which has been proposed by the attention restoration theory to occur when a patient successfully breaks away from routine physical and mental tasks and switches from an effortful, directed attention to an interest-driven one – both of which can be achieved by providing an adequate environment that is stimuli rich, coherently structured and allows for exploration [97]. The ACCT intervention in the hospital might provide such an environment, whereas the paper and pencil intervention at home does not. The subgroup analysis also replicated the sample’s improvement over time in attention ASCS of the complete case analysis. Improvement in attention and memory ASCS predicted depression improvement at follow-up; the more patients improved in attention and memory ASCS, the more they improved in depression. Notice, however, that the directionality of this relationship remains unclear. However, the intercept indicates that there seems to be a negative improvement in depression if no improvement in attention or memory is present. It is known that depression correlates with cognitive deficits, specifically in nonverbal problem solving, verbal and visual memory and attention, and psychomotor speed [55]. Potentially, the improvement in attention or memory through training resulted also in a reduction of depression levels in our sample. Alternatively, the training induced a change in mood, which resulted in cognitive improvement. This subgroup analysis is particularly interesting because, according to our exclusion criteria, patients with mental illness should not have passed the screening process. This result underlines the notion that mental problems often remain undiagnosed or are neglected when assessing the health status of the patient, despite the known impact of depressive mood on cognitive ability, independence, impairment, and handicap [55].

There are several limitations to this study. Firstly, this pilot comprises of a small sample size. More patients would be necessary to confirm the indicated results with adequate power. Also, a larger sample is necessary to check if specific cognitive aspects of the training scenarios influence groups of patients with similar deficit profiles differently. However, the number of neuropsychological tests performed was excessive for the sample size tested. Further, we are aware that the experimental intervention appears to be substantially different from the control intervention in terms of location (hospital versus home) and human interaction (therapist versus, possibly, caregiver). Although a control intervention in the hospital would appear appropriate on methodological grounds, our control condition represents the reality of community-dwelling stroke patients and is, therefore, closer to the “best available” treatment [36]. Besides, the EG did not receive more attention from a therapist than the CG. The patients at the hospital were independently completing their daily tasks, only receiving technical support from the therapist when needed and no performance feedback. However, it cannot be excluded that the different locations might have exposed the patients in the EG to a richer social environment and influenced our results. Hence future studies should test for the potential effect of location on cognition or depression and take it into account when designing their protocols. Further, we were not able to blind the patients and could only partly blind the outcome assessor. This is, unfortunately, a problem frequently encountered in studies evaluating VR-interventions. Nevertheless, we believe that our results support the growing evidence that recovery of cognitive functioning after stroke is possible. Since we were able to train stroke survivors with heterogeneity in cognitive impairment, it fuels the hope that rehabilitation approaches in VR that are grounded on neuroscientific principles of recovery could potentially address co-occurring symptoms even independent of disease or aetiology [98]. Future work should, therefore, test the proposed training paradigm in other patient groups with similar cognitive symptomatology.

## Conclusions

Our stroke rehabilitation approach, called ACCT, was able to adapt the training to the individual cognitive deficit of the patients, and initial results indicated that the training reduced the impairment in two out of four cognitive domains. In addition, a positive change in the mental wellbeing of the patients was observed. This work, therefore, highlights the importance of addressing cognitive domains in conjunction as well as considering the psychological sequelae after a stroke incident.

## Supplementary information

**Additional file 1. Supplementary Material.** The document contains further information on the experimental intervention and the statistical procedure as well as additional **Figures S1** and **S2** and additional **Tables S1** to **S5** not shown but referenced in the main manuscript.

**Additional file 2: Movie S1.** A video showing the intervention in action.

## Data Availability

The datasets used and analysed during the current study are available from the corresponding author on reasonable request.

## Abbreviations

ACCT: Adaptive conjunctive cognitive training
ASCS: Averaged standardized composite score
BI: Barthel Index
CG: Control group
CGD: Control group in depression subgroup analysis
CID: Clinically important difference
CONSORT: Consolidated Standards of Reporting Trials
Corsi B / Corsi F: Corsi Block Tapping Test Backward / Forward
EG: Experimental group
EGD: Experimental group in depression subgroup analysis
FAB: Frontal Assessment Battery
FM-UE: Fugl-Meyer Assessment for the upper extremity
HAM-D: Hamilton Depression Rating Scale
MDC: Minimum detectable change
MMSE: Mini-Mental State Examination
MoCA: Montreal Cognitive Assessment
MRC: Medical Research Council Scale for stroke assessment
RAVLT D / I: Rey Auditory Verbal Learning Test Delayed Recall / Immediate
RGS: Rehabilitation Gaming System
SD: Standard deviation
Star: Star Cancellation Test
TMT A / B: Trail Making Test A / B
VR: Virtual reality
WAIS B / C / F: Wechsler Adult Intelligence Scale-Fourth Edition Digit Span Backward / Digit Symbol Coding / Digit Span Forward
