# Supplementary material for: Adaptive conjunctive cognitive training (ACCT) in virtual reality for chronic stroke patients: a randomized controlled pilot trial
Source: J Neuroeng Rehabil. 2020 Mar 6;17:42. doi: 10.1186/s12984-020-0652-3 (PMC7059385; doi:10.1186/s12984-020-0652-3)
Supplement: Supplementary file 1 — Additional file 1. Supplementary Material. The document contains further information on the experimental intervention and the statistical procedure as well as additional Figures S1 and S2 and additional Tables S1 to S5 not shown but referenced in the main manuscript. [file 12984_2020_652_MOESM1_ESM.pdf]

SUPPLEMENTARY MATERIAL

Adaptive Conjunctive Cognitive Training (ACCT) in Virtual Reality for Chronic Stroke Patients Modulates Cognitive Abilities and Depression: A Randomized Controlled Trial

Martina Maier[1], Belén Rubio Ballester[1], Nuria Leiva Bañuelos[2], Esther Duarte Oller[2,] and Paul F. M. J. Verschure[1,3]

Affiliation: [1]Laboratory of Synthetic, Perceptive, Emotive and Cognitive Systems (SPECS), Institute for Bioengineering of Catalonia (IBEC), The Barcelona Institute of Science and Technology, Barcelona Spain. [2]Rehabilitation Research Group, Institut Hospital del Mar d'Investigacions Mèdiques (IMIM), Physical Medicine and Rehabilitation Department Parc de Salut Mar (Hospital del Mar, Hospital de l'Esperança), Barcelona, Spain. [3]Institució Catalana de Recerca I Estudis Avançats (ICREA), Barcelona, Spain.

Corresponding author: Paul F. M. J. Verschure, Av. d'Eduard Maristany 10-14, 08019 Barcelona, Spain, pverschure@ibecbarcelona.eu

EXPERIMENTAL INTERVENTION

Complex Spheroids

The Complex Spheroids scenario is a basic attention and memory training without automated adaptation of difficulty (Figure 1C), allowing measuring basic cognitive function in the absence of adaptation. It requires the patient to intercept approaching coloured spheres by following a predefined sequence indicated at the top right corner of the screen. The patient must keep the current position of the colour sequence in memory. The spheres can either approach on the left, on the right or on both sides of the workspace that is divided by a white line. It prompts the patient to either focus his attention on one side or divide it to both sides of the screen. Errors in the sequence are indicated with a tone. When the patient completes the sequence correctly three times in a row, he is rewarded with a point, and the sequence changes to a new one.

Star Constellations

The Star Constellations scenario (Figure 1D) supports the training of spatial attention and spatial memory as well as working memory load and memory delayed recall. In a given trial, a star constellation is shown to the patient, and a subset of the stars light up in a sequence. The sequence must be kept in memory and after a delay period reproduced by touching the stars accordingly. Correctly reproducing the sequence rewards the patient with one point for each star in the sequence and lights up the whole constellation. If the patient committed a mistake, the wrongly touched stars are coloured in red. All actions are accompanied by distinct sounds. The difficulty of four task parameters is adapted in this task; 1) there are seven categories of constellations that vary in terms of complexity (simple four-star constellations to complex 13-star ones) and spatial extension (simple centrally concentrated to maximally extended to both sides of the workspace). This parameter trains spatial attention and spatial memory; 2) and 3) the number of stars in a sequence (from 3 to all) and

the time interval between the appearance of the individual stars (ranging from 4 to 2 seconds) aid the training of working memory; 4) the length of the delay period can range from 1 to 5 seconds therefore progressively challenging memory delayed recall.

Quality Controller

The Quality Controller scenario (Figure 1E) aims for training selective, sustained and divided attention, alertness, spatial awareness as well as components of executive functioning like planning, inhibition, and error correction. The patient is presented with two concurring tasks. In the right workspace, doughnuts must be taken out of a fryer when their cooking time ends as indicated by the sound of an alarm clock. If the patient moves his arm over the fryer at the right time, he is rewarded with one point. If he reacts too late or too early, he is penalized with a minus point. In the left workspace, a machine produces candies, that move over a conveyor belt. The type of candy currently produced is indicated in a display on the machine. The patient must spot candies on the conveyor belt that do not match the indicated sample and push them away. For every correctly spotted defective candy, the patient is rewarded with a point. If a non-defective candy is touched, the patient loses a point, and the touched candy lights up red. The difficulty of five task parameter is adapted in this scenario: 1) The speed of the conveyor belt (from 2 meters per second to 5 meters per second). 2) The interval between appearing candies (4 to 2 seconds). These two parameters address speed-of-processing training. 3) The ratio between defective and good candies can change from 8 out of 24 to 1 out of 3 increasing the speed of change in candy type promoting selective and sustained attention; 4) The baking time of the doughnuts (from 30 to 5 seconds) trains inhibition of response. 5) The time given to take them out of the fryer (from 6 seconds to 3 seconds) trains initiation of response. The difficulty parameters of the two tasks adapt independently but the subject have to take care of both tasks at the same time. This promotes divided attention, alerting and problem-solving techniques. Lastly, the spatial layout of the task stimulates spatial awareness.

STATISTICAL PROCEDURE

For the minimization procedure, the following cut-offs scores were considered for creating the strati:

	1st stratum	2nd stratum	3rd stratum	4th stratum
BI (1)	< 51	> 50 and < 76	> 75	Na
Corsi F (2)	< 4	> 3 and < 6	> 5	Na
Corsi B (2)	< 4	> 3 and < 6	> 5	Na
FAB (3)	< 13.7	> 13.6 and < 14.26	> 14.25	Na
FM-UE (4)	< 20	> 19 and < 47	> 46	Na
MoCA (5)	< 11	> 10 and < 21	> 20 and < 26	> 25
MMSE (5)	< 11	> 10 and < 21	> 20 and < 27	> 26
RAVLT I (6)	< 24	> 23 and < 28	> 27 and < 32	> 31
RAVLT D (6)	< 4	> 3 and < 5	> 4 and < 6	> 5
Star (7)	< 44	> 43	Na	Na
TMT A (2)	> 103	< 104 and > 62	< 63	Na
TMT B (2)	> 266	< 267 and > 156	< 157	Na
WAIS F (2)	< 4	4	> 4	Na
WAIS B (2)	< 2	> 1 and < 5	> 4	Na
WAIS C (2)	< 13	> 12 and < 25	> 24	Na

In order to assess how well the task parameters of each training scenario correlate with the scores of the neuropsychological test battery, we calculated first for each patient, daily session and task parameter the maximum difficulty level achieved of all successful trials. For instance, for the speed of the conveyor belt in the Quality Controller scenario, we took for every patient and session the maximum speed level achieved when correct candies where intercepted. We then calculated per task parameter the median over the first week of training and correlated this value with the patient's baseline score of each test in the neuropsychological test battery.

For the primary outcomes and following similar studies in the field (8,9), we created composite scores by taking the individual test scores for each test in the neuropsychological test battery and converting them to standardized z-scores, using the mean and standard deviation (SD) of the normative age-adjusted data for a given test (2,3,6,7). The following scores of each test and corresponding normative data were taken to compute the standardized z-score: Corsi F longest span achieved, age range for norms 62-72 (2), TMT A seconds to complete, age range for norms 62-72 (2), WAIS F max digit range achieved, age range for norms 62-72 years (2), Corsi B longest span achieved, age range for norms 62-72 (2), RAVLT I total recall, age 65 years, male (6) RAVLT D number of recalled words, age 65 years, male (6), WAIS B max range achieved, age range for norms 62-72 (2) TMT B seconds to complete, age range for norms 62-72 (2), WAIS C number of correct substitutions age range for norms 62-72 (2), FAB mean scores, age 60-69, education 6-8 years (3) and Star items detected, patient no cognitive impairment BIT (7). The resulting z-scores were averaged to obtain the patient's average standardized composite score (ASCS) for a given domain. To obtain a measurement of generalized cognitive functioning, we took the median of the patient's ASCS for each domain. To obtain an overview with regarding to impairment level, each patient's ASCS per domain was stratified into 'no impairment' (higher than normative data), 'mild' (within -1 SD from normative data), 'moderate' (between -1 and -2 SD from normative data) and 'severe' (more than -2 SD from normative data). We adopted a finer gradient of impairment level as classically reported (10,11). To evaluate the change, we first used Spearman's correlation to evaluate the relationship between all ASCS at the three time points. We then compared the ASCS scores over time using Friedman's ANOVA test statistic (χ_F^2) within each group. Post-hoc analysis was performed using Wilcoxon's sign rank test (T). We calculated the improvement after treatment (T1 – T0) and at follow-up (T2 – T0) and first calculated the significant difference from 0 per group using a Wilcoxon's sign rank test (T) and then the difference between groups using the Wilcoxon's rank-sum test (W_s). The same procedure was applied to the secondary outcomes. Although the incidents that led to drop-outs and therefore missing data seem unrelated to the study, we compare the outcomes of complete-case analysis to an intention to treat analysis, with imputed data (last observation carried forward), to determine the sensitivity of the results. Significant results were only accepted when confirmed by both analyses. Lastly, we analysed the ASCS of those participants for which the HAM-D was obtained (EG=11, CG=10). In addition to the statistical procedure as described above, we used linear regression to examine which cognitive impairment contributed the most to the depression level observed. With the HM-D score as the response variable, the domain ASCS was included in the model as a predictor. We used MATLAB R2017b for all statistical analysis, except for the regression where we used the lm-package in R version 3.5.0. The minimization procedure was processed through a custom-made MATLAB-script which was based on the open-source software OxMaR (12). The data are presented as frequencies with percentages, means with SD, and medians with the 25th and 75th percentile, as appropriate.

SUPPLEMENTARY FIGURES

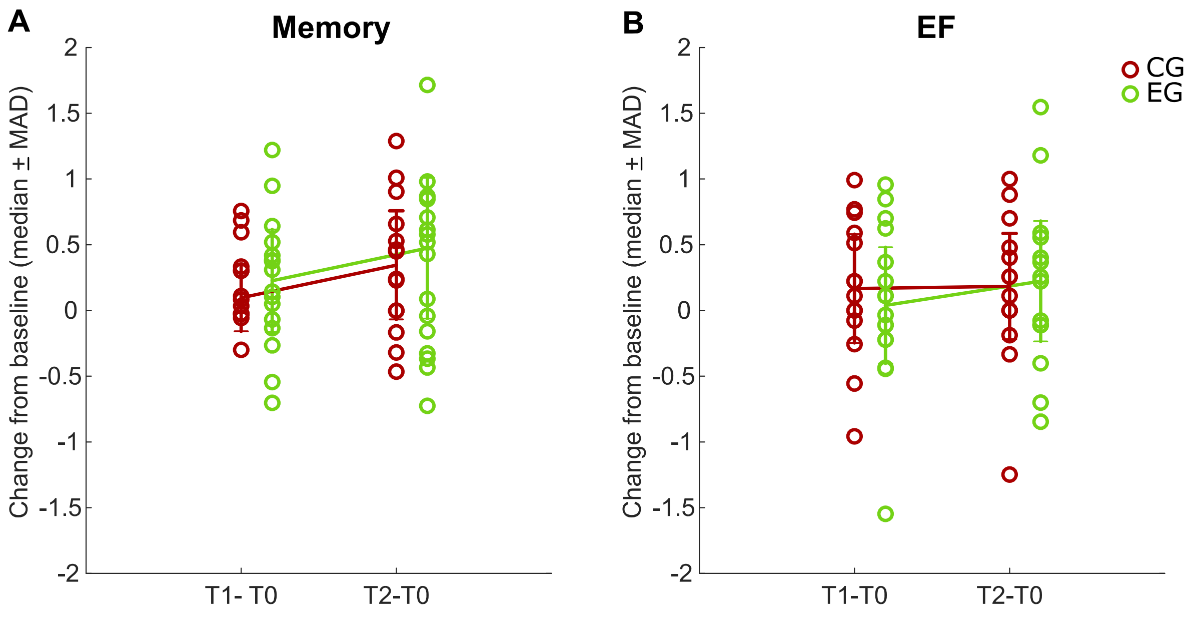

Figure S1. ASCS scores for memory and executive functioning. Change in (A) Memory ASCS and (B) EF ASCS from baseline to after treatment (T1) and to follow-up (T2) for the experimental group (EG, green) and control group (CG, red). Error bars indicate median absolute deviation (MAD) for each group. The individual data for each subject is indicated with dots.

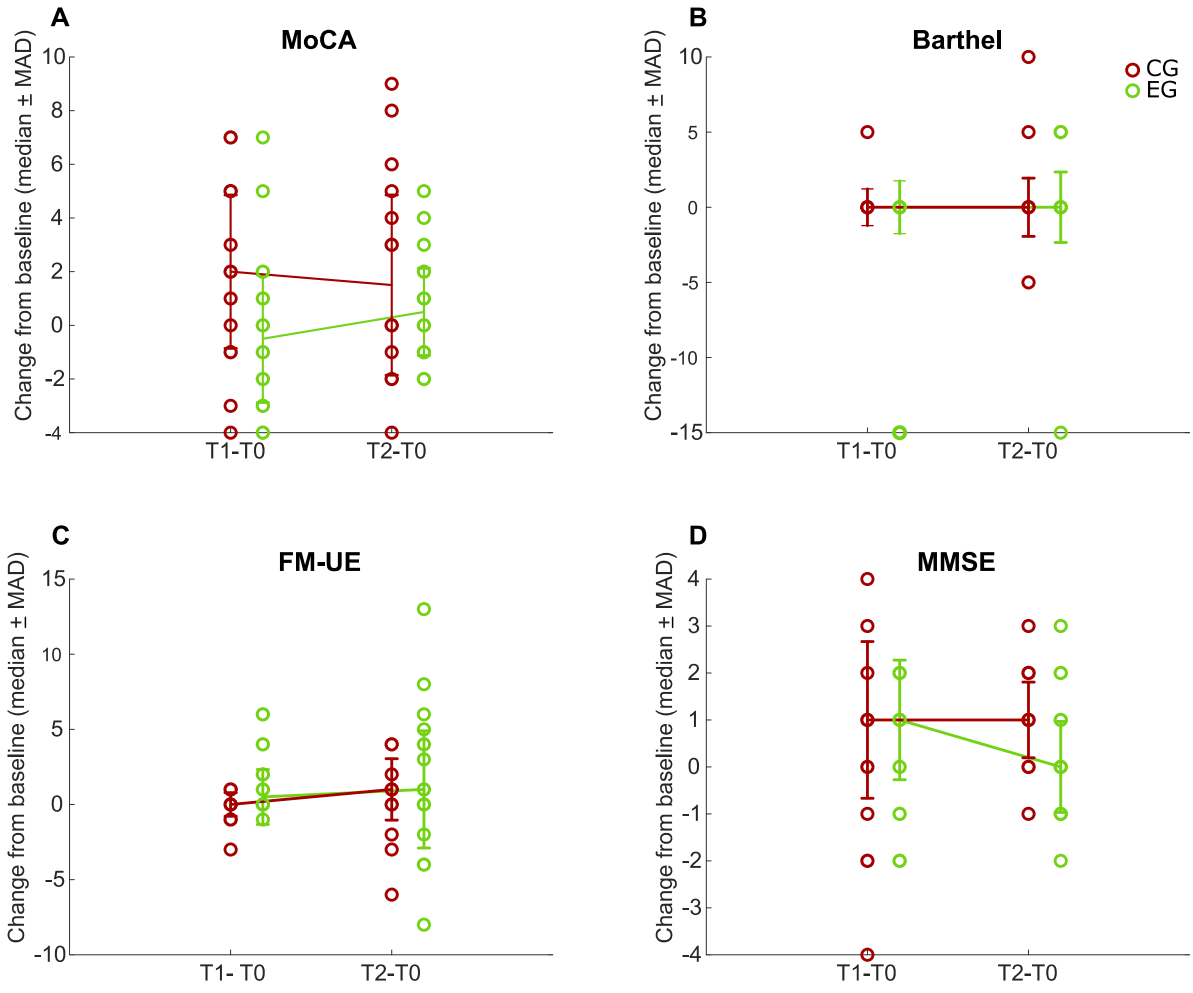

Figure S2. Secondary outcome measurements. Change in (A) MoCA, (B) BI and (C) FM-UE and (D) MMSE from baseline to after treatment (T1) and to follow-up (T2) for the experimental group (EG, green) and control group (CG, red). Error bars indicate median absolute deviation (MAD) for each group. The individual data for each subject is indicated with dots.

SUPPLEMENTARY TABLES

Table S1. ASCS complete case analysis and last observation carried forward analysis. Evaluation of within-group change over time and improvement T1-T0 and T2 -T0, as well as between-group differences at these time points.

ASCS		Baseline (T0)	After treatment (T1)	Follow-up (T2)	Friedman's ANOVA		T1-T0	T2-T0
		Mean (SD) – Median [2.5th – 97.5th percentile]						
**Attention**								
CC	EG	-0.35 (0.88) – -0.28 [-2.11 – 1.22]	-0.13 (0.94) – -0.17 [-1.67 – 1.33]	**0.06 (0.92) – 0.17 [-1.44 – 1.67] ***	$\chi_F^2(2)$	**.01**	0.22 (0.39) – 0.17 [-0.44 – 0.89]	0.41 (0.46) – 0.44 [-0.44 – 1.33]
	CG	-0.16 (0.83) – 0.11 [-1.78 – 0.89]	0.02 (0.80) – 0.28 [-1.78 – 1.00]	0.03 (0.92) – 0.22 [-1.67 – 1.67]	$\chi_F^2(2)$	.25	0.17 (0.50) – 0.17 [-0.78 – 1.00]	0.19 (0.32) – 0.17 [-0.44 – 0.78]
between-group		W_s .39				W_s	.80	.21
LOCF	EG	-0.44 (0.84) – -0.33 [-2.11 – 1.22]	**-0.23 (0.91) – -0.33 [-1.67 – 1.33] ***	**-0.08 (0.91) – 0 [-1.44 – 1.67] ****	$\chi_F^2(2)$	**.00**	0.20 (0.36) – 0.11 [-0.44 – 0.89]	0.36 (0.44) – 0.44 [-0.44 – 1.33]
	CG	-0.35 (0.90) – -0.22 [-2.00 – 0.89]	-0.23 (0.90) – -0.11 [-2.00 – 1.00]	-0.22 (0.98) – -0.11 [-2.00 – 1.67]	$\chi_F^2(2)$	.42	0.11 (0.44) – 0.00 [-0.78 – 1.00]	0.12 (0.30) – 0.11 [-0.44 – 0.78]
between-group		W_s .54				W_s	.36	.09
**Memory**								
CC	EG	-0.76 (0.69) – -0.57 [-2.27 – 0.05]	-0.54 (0.91) – -0.31 [-2.17 – 0.76]	-0.43 (0.91) – -0.30 [-2.19 – 0.89]	$\chi_F^2(2)$	.30	0.21 (0.50) – 0.23 [-0.70 – 1.22]	0.33 (0.65) – 0.47 [-0.73 – 1.71]
	CG	-0.72 (0.82) – -0.54 [-2.38 – 0.40]	**-0.52 (0.73) – -0.44 [-1.78 – 0.56] ***	-0.37 (0.83) – -0.52 [-1.37 – 1.52]	$\chi_F^2(2)$	.42	0.20 (0.31) – 0.10 [-0.30 – 0.76]	0.34 (0.51) – 0.34 [-0.47 – 1.29]
between-group		W_s .85				W_s	.82	.92
LOCF	EG	-0.93 (0.76) – -0.85 [-2.27 – 0.05]	-0.71 (0.95) – -0.57 [-2.22 – 0.76]	**-0.61 (0.96) – -0.45 [-2.22 – 0.89] ***	$\chi_F^2(2)$	.20	0.22 (0.49) – 0.14 [-0.70 – 1.22]	0.32 (0.61) – 0.43 [-0.73 – 1.71]

	CG	-0.83 (0.78) – -0.78 [-3.21 – 1.79]	**-0.66 (0.75) – -0.70 [-2.24 – 0.56] ***	**-0.55 (0.84) – -0.70 [-2.24 – 1.52] ***	$\chi^2_F(2)$	.18		0.17 (0.28) – 0.08 [-0.30 – 0.76]	0.28 (0.46) – 0.23 [-0.47 – 1.29]
between-group		W_s .70					W_s	.64	.91
**EF**									
CC	EG	-0.34 (1.01) – -0.335 [-1.64 – 1.32]	-0.29 (1.18) – -0.38 [-2.09 – 2.02]	-0.15 (1.19) – 0.15 [-1.97 – 1.68]	$\chi^2_F(2)$	.43		0.05 (0.61) – 0.04 [-1.55 – 0.96]	0.19 (0.62) – 0.22 [-0.85 – 1.55]
	CG	-0.45 (1.38) – -0.27 [-2.67 – 1.79]	-0.28 (1.33) – -0.02 [-2.60 – 2.02]	-0.28 (1.40) – -0.21 [-2.60 – 1.91]	$\chi^2_F(2)$	.47		0.17 (0.54) – 0.17 [-0.30 – 0.76]	0.17 (0.56) – 0.18 [-1.25 – 1.00]
between-group		W_s .92					W_s	.57	.79
LOCF	EG	-0.53 (1.08) – -0.50 [-2.70 – 1.32]	-0.49 (1.23) – -0.54 [-2.70 – 2.02]	-0.38 (1.25) – -0.09 [-2.70 – 1.68]	$\chi^2_F(2)$	.47		0.04 (0.56) – 0.00 [-1.55 – 0.96]	0.15 (0.57) – 0.00 [-0.85 – 1.55]
	CG	-0.69 (1.40) – -0.69 [-3.21 – 1.79]	-0.59 (1.39) – -0.38 [-3.21 – 2.02]	-0.59 (1.44) – -0.38 [-3.21 – 1.91]	$\chi^2_F(2)$	.85		0.10 (0.48) – 0.00 [-0.96 – 0.99]	0.10 (0.50) – 0.00 [-1.25 – 1.00]
between-group		W_s .92					W_s	.66	.88
**SA**									
CC	EG	-2.88 (6.57) – -0.39 [-25.17 – 0.50]	**-0.67 (3.95) – 0.50 [-15.43 – 0.50] ***	**0.33 (0.36) – 0.50 [-0.39 – 0.50] ***	$\chi^2_F(2)$	**.00**		2.21 (6.55) – 0.88 [-6.19 – 24.78]	3.21 (6.57) – 0.88 [-0.88 – 25.66]
	CG	-0.58 (1.44) – 0.05 [-3.93 – 0.50]	-0.20 (1.44) – 0.50 [-4.81 - 50]	-0.52 (1.90) – 0.50 [-6.68 – 0.50]	$\chi^2_F(2)$	.53		0.38 (1.08) – 0.00 [-0.88 – 2.65]	0.06 (1.95) – 0.00 [-5.31 – 2.65]
between-group		W_s .45					W_s	.24	.10
LOCF	EG	-2.53 (6.05) – -0.39 [-25.17 – 0.50]	**-0.58 (3.62) – 0.50 [-15.43 – 0.50] ***	**0.26 (0.40) – 0.50 [-0.39 – 0.50] ****	$\chi^2_F(2)$	**.00**		1.96 (6.02) – 0.88 [-6.18 – 24.78]	2.79 (6.09) – 0.88 [-0.88 – 25.66]
	CG	-0.81 (1.94) – 0.50 [-6.58 – 0.50]	-0.44 (1.95) – 0.50 [-6.58 – 0.50]	-0.67 (2.19) – 0.50 [-6.58 – 0.50]	$\chi^2_F(2)$	.37		0.37 (0.99) – 0.00 [-0.88 – 2.65]	0.10 (0.00) – 0.00 [-5.31 – 2.65]

between-group		W_s .35			W_s		.18	.07
**GCF**								
CC	EG	-0.56 (0.79) – -0.44 [-1.92 – 0.39]	**-0.20 (0.80) – -0.10 [-1.64 – 0.91] ****	**-0.12 (0.83) – 0.25 [-1.56 – 0.99] ****	$\chi^2_F(2)$	**.00**	0.36 (0.36) – 0.34 [-0.06 – 0.90]	0.44 (0.42) – 0.54 [-0.56 – 1.17]
	CG	-0.38 (0.90) – 0.00 [-2.00 – 0.69]	-0.17 (0.81) – 0.10 [-1.93 – 0.75]	-0.16 (0.94) – 0.06 [-1.98 – 1.59]	$\chi^2_F(2)$	.93	0.21 (0.45) – 0.13 [-0.31 – 1.27]	0.22 (0.37) – 0.14 [-0.22 – 0.90]
between-group		W_s .49			W_s		.12	.12
LOCF	EG	-0.66 (0.78) – -0.58 [-1.92 – 0.39]	**-0.33 (0.82) – -0.26 [-1.72 – 0.91] ****	**-0.27 (0.86) – -0.14 [-1.72 – 0.99] ****	$\chi^2_F(2)$	**.00**	0.33 (0.30) – 0.32 [-0.06 - 0.90]	0.39 (0.41) – 0.42 [-0.56 – 1.17]
	CG	-0.56 (0.95) – -0.21 [-2.17 – 0.69]	-0.41 (0.92) – -0.16 [-2.17 – 0.75]	-0.40 (1.01) – -0.25 [-2.17 – 1.59]	$\chi^2_F(2)$	.94	0.15 (0.40) – 0.00 [-0.31 – 1.27]	0.17 (0.33) – 0.00 [-0.22 – 0.90]
between-group		W_s .73			W_s		**.04**	**.04**

For within-group change over time, we used Friedman's ANOVA test statistic, for within-group post hoc analysis of the differences Wilcoxon's sign rank test, and between-group Wilcoxon's rank-sum test. Significant comparisons with respect to baseline are indicated with * for *p*-values < .05 and ** for *p*-values < .01. *ASCS* average standardized composite score, *CC* complete case analysis, *CG* control group, *GCF* generalized cognitive functioning, *EF* executive functioning, *EG* experimental group, *LOCF* last observation carried forward, SA spatial awareness, χ^2_F Friedman's ANOVA test statistic, *T* Wilcoxon's sign rank test, *Ws* Wilcoxon's rank-sum test

Table S2. Neuropsychological test battery complete case analysis and last observation carried forward analysis. Evaluation of within-group change over time and improvement T1 - T0 and T2 - T0, as well as between-group differences at baseline, T1 - T0, and T2 -T0.

Scale		Baseline (T0)	After treatment (T1)	Follow-up (T2)	Friedman's ANOVA	T1-T0	T2-T0
		Mean (SD) – Median [2.5th – 97.5th percentile]					
**Corsi F**							
CC	EG	5.56 (1.59) – 5.50 [3.00 – 9.00]	6.06 (2.08) – 6.00 [3.00 – 9.00]	**6.81 (2.17) – 6.00 [4.00 – 11.00]***	χ^2_F (2) .13	0.50 (1.59) – 0.50 [-3.00 – 4.00]	1.25 (1.91) – 2.00 [-2.00 – 5.00]
	CG	5.57 (1.60) – 6.00 [3.00 – 9.00]	5.86 (1.46) – 5.00 [4.00 – 8.00]	6.14 (1.51) – 6.00 [4.00 – 9.00]	χ^2_F (2) .40	0.29 (2.05) – 0.50 [-4.00 – 3.00]	0.57 (2.03) – 1.00 [-5.00 – 3.00]
between-group		*Ws* .88			*Ws*	.87	.45
LOCF	EG	5.32 (1.60) – 5.00 [3 – 9]	5.84 (1.98) – 6.00 [3.00 – 9.00]	**6.47 (2.14) – 6.00 [4.00 – 11.00]****	χ^2_F (2) .08	0.53 (1.50) – 0.00[-3.00 – 4.00]	1.16 (1.80) – 2.00 [-2.00 – 5.00]
	CG	5.37 (1.54) – 5.00 [3 – 9]	5.53 (1.43) – 5.00 [4.00 – 8.00]	5.74 (1.52) – 6.00 [4.00 – 9.00]	χ^2_F (2) .61	0.16 (1.77) – 0.00 [-4.00 – 3.00]	0.37 (1.77) – 0.00 [-5.00 – 3.00]
between-group		*Ws* .86			*Ws*	.36	.09
**Corsi B**							
CC	EG	4.31 (1.78) – 5.00 [1.00 – 6.00]	5.00 (2.03) – 5.00 [2.00 – 10.00]	4.88 (1.86) – 5.00 [2.00 – 4.86]	χ^2_F (2) .59	0.69 (1.58) – 0.00 [-2.00 – 4.00]	0.56 (1.67) – 0.50 [-2.00 – 4.00]
	CG	4.57 (2.10) – 5.00 [2.00 – 9.00]	4.93 (2.20) – 5.00 [2.00 – 9.00]	4.86 (1.46) – 5.00 [2.00 – 7.00]	χ^2_F (2) .85	0.36 (1.22) – 0.00 [-1.00 – 3.00]	0.29 (1.73) – 0.00 [-2.00 – 3.00]
between-group		*Ws* .95			*Ws*	.54	.69

LOCF	EG	4.21 (1.72) – 5.00 [1 – 6]	4.74 (2.02) – 5.00 [2.00 – 10.00]	4.63 (1.86) – 5.00 [2.00 – 8.00]	χ_F^2 (2)	.79		0.53 (1.50) – 0.00 [-2.00 – 4.00]	0.42 (1.57) – 0.00 [-2.00 – 4.00]
	CG	4.47 (1.87) – 4.00 [2 – 9]	4.74 (1.91) – 5.00 [2.00 – 9.00]	4.68 (1.29) – 4.00 [2.00 – 7.00]	χ_F^2 (2)	.67		0.26 (1.10) – 0.00 [-1.00 – 3.00]	0.21 (1.51) – 0.00 [-2.00 – 3.00]
between-group		*Ws* .91					W_s	.63	.74
**FAB**									
CC	EG	16.38 (1.45) – 16.50 [14.00 – 18.00]	16.44 (2) – 17.00 [11.00 – 18.00]	16.75 (1.88) – 17.50 [12.00 – 18.00]	χ_F^2 (2)	.25		0.06 (1.48) – 0.00 [-3.00 – 2.00]	0.38 (1.36) – 0.00 [-2.00 – 3.00]
	CG	16.00 (2.42) – 17.00 [11.00 – 18.00]	16.43 (1.65) – 17.00 [13.00 – 18.00]	16.43 (1.95) – 17.50 [13.00 – 18.00]	χ_F^2 (2)	.22		0.43 (1.45) – 0.00 [-2.00 – 3.00]	0.43 (1.55) – 0.50 [-4.00 – 3.00]
between-group		*Ws* 1					W_s	.76	.73
LOCF	EG	15.95 (1.99) – 16.00 [10 – 18]	15.95 (2.39) – 17.00 [10.00 – 18.00]	16.21 (2.37) – 17.00 [10.00 – 18.00]	χ_F^2 (2)	.40		0.00 (1.37) – 0.00 [-3.00 – 2.00]	0.26 (1.28) – 0.00 [-2.00 – 3.00]
	CG	15.68 (2.87) – 17.00 [8 -–18]	15.89 (2.45) – 17.00 [8.00 – 18.00]	15.89 (2.60) – 17.00 [8.00 – 18.00]	χ_F^2 (2)	.67		0.21 (1.32) – 0.00 [-2.00 – 3.00]	0.21 (1.40) – 0.00 [-4.00 – 3.00]
between-group		*Ws* 0.81					W_s	1	.94
**RAVLT D**									
CC	EG	4.69 (2.70) – 4.00 [0.00 – 10.00]	5.25 (3.15) – 4.50 [0.00 – 11.00]	5.63 (2.73) – 6.00 [1.00 – 11.00]	χ_F^2 (2)	.48		0.56 (1.90) – 0.00 [-2.00 – 5.00]	0.94 (2.46) – 0.50 [-3.00 – 6.00]
	CG	5.21 (2.99) – 5.50 [0.00 – 10.00]	6.21 (2.49) – 6.00 [2.00 – 10.00]	6.50 (2.47) – 6.00 [3.00 – 13.00]	χ_F^2 (2)	.54		1.00 (1.88) – 0.00 [-2.00 – 5.00]	1.29 (2.76) – 0.50 [-2.00 – 6.00]
between-group		*Ws* .50					W_s	.43	.85

LOCF	EG	4.16 (2.77) –<br>4.00 [0 – 10]	4.84 (3.11) –<br>4.00 [0.00 – 11.00]	5.16 (2.81) –<br>6.00 [1.00 – 11.00]	χ_F^2 (2)	.36		0.68 (1.92) –<br>0.00 [-2.00 – 5.00]	1.00 (2.38) –<br>0.00 [-3.00 – 6.00]
	CG	4.95 (2.74) –<br>5.00 [0 – 10]	**5.84 (2.43) –<br>6.00 [1.00 – 10.00]***	6.05 (2.46) –<br>6.00 [1.00 – 13.00]	χ_F^2 (2)	.23		0.89 (1.66) –<br>0.00 [-2.00 – 5.00]	1.11 (2.40) –<br>0.00 [-2.00 – 6.00]
between-group		*Ws* .31					*Ws*	.49	.99
**RAVLT I**									
CC	EG	32.38 (9.70) –<br>34.00 [10.00 – 44.00]	32.69 (10.71) –<br>34.50 [8.00 – 48.00]	35.06 (11.37) –<br>36.00 [7.00 – 49.00]	χ_F^2 (2)	.53		0.31 (5.62) –<br>-0.50 [-7.00 – 14.00]	2.69 (6.59) –<br>2.00 [-6.00 – 15.00]
	CG	32.57 (10.82) –<br>34.00 [14.00 – 47.00]	32.64 (9.34) –<br>32.50 [17.00 – 45.00]	35.57 (9.74) –<br>38.50 [20.00 – 54.00]	χ_F^2 (2)	.17		0.07 (5.48) –<br>0.00 [-7.00 – 11.00]	3.00 (6.04) –<br>3.00 [-10.00 – 13.00]
between-group		*Ws* .82					*Ws*	.93	.71
LOCF	EG	30.53 (9.9) –<br>33.00 [10 – 44]	31.63 (10.68) –<br>31.00 [8.00 – 48.00]	33.63 (11.46) –<br>35.00 [7.00 – 49.00]	χ_F^2 (2)	.45		1.11 (6.27) –<br>0.00 [-7.00 – 16.00]	3.11 (6.83) –<br>2.00 [-6.00 – 16.00]
	CG	30.21 (10.49) –<br>29.00 [13 – 47]	30.79 (9.34) –<br>30.00 [13.00 – 45.00]	**32.95 (10.14) –<br>30.00 [13.00 – 54.00]***	χ_F^2 (2)	.09		0.58 (4.91) –<br>0.00 [-7.00 – 11.00]	2.74 (5.31) –<br>3.00 [-10.00 – 13.00]
between-group		*Ws* .98					*Ws*	.98	.80
**Star**									
CC	EG	50.19 (7.42) –<br>53.00 [25.00 – 54.00]	**52.69 (4.47) –<br>54.00 [36.00 – 54.00]***	**53.81 (0.40) –<br>54.00 [53.00 – 54.00]***	χ_F^2 (2)	**.00**		2.50 (7.40) –<br>1.00 [-7.00 – 28.00]	3.63 (7.43) –<br>1.00 [-1.00 – 29.00]
	CG	32.57 (1.63) –<br>53.50 [49.00 – 54.00]	53.21 (1.63) –<br>54.00 [48.00 – 54.00]	52.86 (2.14) –<br>54.00 [46.00 – 54.00]	χ_F^2 (2)	.53		0.43 (1.22) –<br>0.00 [-1.00 – 3.00]	0.07 (2.20) –<br>0.00 [-6.00 – 3.00]
between-group		*Ws* .45					*Ws*	.23	.10

LOCF	EG	50.58 (6.84) – 53.00 [25 – 54]	**52.79 (4.09) – 54.00 [36.00 – 54.00]***	**53.74 (0.45) – 54.00 [53.00 – 54.00]****	χ^2_F (2)	**.00**	2.21 (6.80) – 1.00 [-7.00 – 28.00]	3.16 (6.88) – 1.00 [-1.00 – 29.00]
	CG	52.53 (2.20) – 54.00 [46 – 54]	52.95 (2.20) – 54.00 [46.00 – 54.00]	52.68 (2.47) – 54.00 [46.00 – 54.00]	χ^2_F (2)	.37	0.42 (1.12) – 0.00 [-1.00 – 3.00]	0.16 (1.92) – 0.00 [-6.00 – 3.00]
between-group		*Ws* .35				*Ws*	.18	.08
**TMT A**								
CC	EG	74.06 (39.83) – 66.00 [31.00 – 154.00]	73.69 (40.13) – 73.50 [22.00 – 150.00]	**63.19 (32.65) – 52.00 [26.00 – 132.00]***	χ^2_F (2)	.26	0.38 (13.80) – 0.50 [-28.00 – 34.00]	10.88 (23.26) – 8.50 [-48.00 – 50.00]
	CG	75.86 (49.98) – 65.00 [29.00 – 176.00]	**58.36 (27.91) – 50.50 [29.00 - 126.00]***	72.43 (57.57) – 55.50 [20.00 – 240.00]	χ^2_F (2)	.14	17.50 (27.32) – 10.50 [-16.00 – 3.00]	3.43 (29.11) – 7.00 [-75.00 – 43.00]
between-group		*Ws* .72				*Ws*	**.03**	.52
LOCF	EG	76.79 (37.89) – 66.00 [31 – 154]	75.11 (37.60) – 78.00 [22.00 – 150.00]	**66.26 (31.66) – 62.00 [26.00 – 132.00]***	χ^2_F (2)	.18	1.68 (13.91) – 0.00 [-28.00 – 34.00]	10.53 (21.83) – 7.00 [-48.00 – 50.00]
	CG	79.68 (47.37) – 66.00 [29 – 176]	68.05 (34.99) – 56.00 [29.00 – 159.00]	78.42 (53.70) – 62.00 [20.00 – 240.00]	χ^2_F (2)	.45	11.63 (25.77) – 7.00 [-23.00 – 93.00]	1.26 (25.47) – 0.00 [-75.00 – 43.00]
between-group		*Ws* .93				*Ws*	.22	.22
**TMT B**								
CC	EG	228.75 (129.82) – 180.50 [70.00 – 402.00]	228.44 (136.98) – 185.00 [52.00 - 402.00]	211.25 (130.59) – 169.00 [48.00 – 402.00]	χ^2_F (2)	.26	0.31 (91.27) – 1.00 [-264.00 – 142.00]	17.50 (90.31) – 4.00 [-192.00 – 229.00]
	CG	212.36 (140.95) – 177.00 [44.00 – 402.00]	209.21 (138.36) – 178.50 [51.00 - 402.00]	225.29 (152.69) – 202.00 [38.00 – 402.00]	χ^2_F (2)	.90	3.14 (35.33) – 0.00 [-80.00 – 75.00]	-12.93 (44.37) – 0.00 [-126.00 – 55.00]
between-group		*Ws* .60				*Ws*	.59	.20

LOCF	EG	242.42 (132.66) – 198.00 [70 – 402]	242.16 (138.56) – 200.00 [52.00 – 402.00]	227.68 (135.04) – 173.00 [48.00 – 402.00]	χ^2_F (2)	.26	0.26 (83.32) – 0.00 [-264.00 – 142.00]	14.74 (82.70) – 0.00 [-192.00 – 229.00]
	CG	241.26 (137.47) – 242.00 [44 – 402]	237.84 (136.67) – 219.00 [51.00 – 402.00]	249.68 (145.01) – 267.00 [38.00 – 402.00]	χ^2_F (2)	.74	3.42 (30.35) – 0.00 [-80.00 – 75.00]	-8.42 (38.75) – 0.00 [-126.00 – 55.00]
between-group		*Ws* .86				W_s	.66	.31
**WAIS B**								
CC	EG	3.44 (0.73) – 3.00 [2.00 – 5.00]	3.81 (1.38) – 4.00 [2.00 – 7.00]	3.81 (1.11) – 4.00 [2.00 – 6.00]	χ^2_F (2)	.36	0.38 (1.15) – 0.00 [-1.00 – 3.00]	0.38 (1.02) – 0.00 [-1.00 – 3.00]
	CG	3.36 (1.22) – 3.50 [2.00 – 5.00]	3.43 (1.02) – 3.00 [2.00 – 5.00]	3.64 (1.08) – 3.50 [2.00 – 5.00]	χ^2_F (2)	.22	0.07 (0.92) – 0.00 [-2.00 – 1.00]	0.29 (0.91) – 0.00 [-2.00 – 2.00]
between-group		*Ws* .85				W_s	.63	.98
LOCF	EG	3.26 (0.81) – 3.00 [2 – 5]	3.58 (1.39) – 3.00 [2.00 – 7.00]	3.58 (1.17) – 4.00 [2.00 – 6.00]	χ^2_F (2)	.36	0.32 (1.06) – 0.00 [-1.00 – 3.00]	0.32 (0.95) – 0.00 [-1.00 – 3.00]
	CG	3.37 (1.16) – 3.00 [2 – 5]	3.42 (1.02) – 3.00 [2.00 – 5.00]	3.58 (1.07) – 3.00 [2.00 – 5.00]	χ^2_F (2)	.22	0.05 (0.78) – 0.00 [-2.00 – 1.00]	0.21 (0.79) – 0.00 [-2.00 – 2.00]
between-group		*Ws* .83				W_s	.62	.88
**WAIS F**								
CC	EG	4.88 (1.26) – 5.00 [3.00 – 7.00]	5.19 (1.28) – 5.00 [3.00 – 7.00]	5.13 (1.31) – 5.00 [3.00 – 8.00]	χ^2_F (2)	.17	0.31 (0.60) – 0.00 [-1.00 – 1.00]	0.25 (0.86) – 0.00 [-1.00 – 3.00]
	CG	5.36 (1.08) – 5.00 [3.00 – 7.00]	5.36 (1.15) – 5.50 [3.00 – 7.00]	5.5 (1.29) – 5.00 [3.00 – 8.00]	χ^2_F (2)	.71	0.00 (0.88) – 0.00 [-1.00 – 2.00]	0.14 (0.77) – 0.00 [-1.00 – 2.00]
between-group		*Ws* .26				W_s	.18	.82

LOCF	EG	4.84 (1.17) – 5.00 [3 – 7]	5.05 (1.22) – 5.00 [3.00 – 7.00]	5.00 (1.25) – 5.00 [3.00 – 8.00]	χ_F^2 (2)	.37	0.21 (0.63) – 0.00 [-1.00 – 1.00]	0.16 (0.83) – 0.00 [-1.00 – 3.00]
	CG	5.11 (1.20) – 5.00 [3 – 7]	5.10 (1.24) – 5.00 [3.00 – 7.00]	5.75 (5.21) – 5.00 [3.00 – 8.00]	χ_F^2 (2)	.71	0.00 (0.75) – 0.00 [-1.00 – 2.00]	0.11 (0.66) – 0.00 [-1.00 – 2.00]
between-group		*Ws* .39			W_s		.24	1
**WAIS C**								
CC	EG	29.25 (13.32) – 27.00 [10.00 – 59.00]	30.06 (13.43) – 29.00 [8.00 – 59.00]	29.69 (14.30) – 29.50 [5.00 – 54.00]	χ_F^2 (2)	.26	0.81 (5.09) – 2.50 [-13.00 – 6.00]	0.44 (6.90) – -0.50 [-8.00 – 17.00]
	CG	26.29 (15.13) – 26.00 [5.00 – 57.00]	27.29 (16.51) – 25.00 [7.00 – 61.00]	26.86 (14.34) – 27.50 [5.00 – 54.00]	χ_F^2 (2)	.44	1.00 (4.76) – 1.50 [-7.00 – 10.00]	0.57 (4.99) – 2.00 [-9.00 – 8.00]
between-group		*Ws* .53			W_s		.77	.43
LOCF	EG	28.21 (13.40) – 26.00 [9 – 59]	29.10 (13.75) – 26.00 [8.00 – 59.00]	28.79 (14.44) – 29.00 [5.00 – 54.00]	χ_F^2 (2)	.22	0.89 (4.71) – 2.00 [-13.00 – 6.00]	0.58 (6.35) – 0.00 [-8.00 – 17.00]
	CG	24.32 (14.03) – 25.00 [5 – 57]	25.16 (15.26) – 24.00 [6.00 – 61.00]	24.84 (13.53) – 25.00 [5.00 – 54.00]	χ_F^2 (2)	.48	0.84 (4.39) – 0.00 [-7.00 – 10.00]	0.53 (4.56) – 0.00 [-9.00 – 8.00]
between-group		*Ws* .35			W_s		.62	.52

For change over time, we used Friedman's ANOVA test statistic, for within-group post hoc analysis of the differences Wilcoxon's sign rank test, and between-group Wilcoxon's rank-sum test. Significant comparisons with respect to baseline are indicated with * for p-values < .05 and ** for p-values < .01. *CC* complete case analysis, *CG* control group, *Corsi B* Corsi Block Tapping Test Backward, *Corsi F* Corsi Block Tapping Test Forward, *EG* experimental group, *FAB* Frontal Assessment Battery, *LOCF* last observation carried forward, *RAVLT* Rey Auditory Verbal Learning Test, *RAVLT I* RAVLT Immediate, *RAVLT D* RAVLT Delayed Recall, *Star* Star Cancellation Test, *TMT A* Trail Making Test A, *TMT B* Trail Making Test B, *WAIS* Wechsler Adult Intelligence Scale IV, *WAIS F* WAIS Digit Span Forward, *WAIS B* WAIS Backward, *WAIS C* WAIS Digit Symbol Coding, χ_F^2 Friedman's ANOVA test statistic, *T* Wilcoxon's sign rank test, *Ws* Wilcoxon's rank-sum test

Table S3. Secondary outcomes of complete case analysis and last observation carried forward analysis. Clinical scales at baseline, after treatment and follow-up. Evaluation of within-group change over time and improvement T1 - T0 and T2 - T0, as well as between-group differences at these time points.

Scale		Baseline (T0)	After treatment (T1)	Follow-up (T2)	Friedman's ANOVA	T1-T0	T2-T0
		Mean (SD) – Median [97.5th – 2.5th percentile]					
**MoCA**							
CC	EG	21.05 (2.71) – 21.50 [16.00 – 25.00]	21.38 (4.11) – 21.00 [13.00 – 29.00]	22.25 (2.82) – 23.00 [17.00 – 25.00]	$\chi^2_F(2)$ .70	-0.13 (3.10) – -0.50 [-4.00 – 7.00]	0.75 (2.05) – 0.50 [-2.00 – 5.00]
	CG	20.21 (4.04) – 21.00 [12.00 – 25.00]	22.21 (3.68) – 23.50 [15.00 – 28.00]	22.29 (3.73) – 22.00 [16.00 – 30.00]	$\chi^2_F(2)$ .31	2.00 (3.53) – 2.00 [-4.00 – 7.00]	2.07 (3.97) – 1.50 [-4.00 – 9.00]
between-group		W_s .46			W_s	.09	.46
LOCF	EG	20.32 (3.92) – 21.00 [12.00 – 25.00]	20.74 (5.09) – 21.00 [12.00 – 29.00]	21.47 (4.41) – 23.00 [12.00 – 28.00]	$\chi^2_F(2)$ .65	0.42 (3.66) – 0.00 [-4.00 – 10.00]	1.16 (2.85) – 0.00 [-2.00 – 10.00]
	CG	20.05 (3.79) – 20.00 [12.00 – 25.00]	**21.79 (3.69) – 23.00 [15.00 – 28.00] ***	21.84 (3.73) – 22.00 [16.00 – 30.00]	$\chi^2_F(2)$ .35	1.74 (3.31) – 1.00 [-4.00 – 7.00]	1.79 (3.66) – 0.00 [-4.00 – 9.00]
between-group		W_s .76			W_s	.19	.77
**BI**							
CC	EG	95.31 (7.18) – 100.00 [80.00 – 100.00]	94.38 (9.29) – 100.00 [70.00 – 100.00	95.63 (8.92) – 100.00 [70.00 – 100.00]	$\chi^2_F(2)$ .07	-0.94 (3.75) – 0.00 [-15.00 – 0.00]	0.31 (4.64) – 0.00 [-15.00 – 5.00]
	CG	87.86 (21.64) – 100.00 [20.00 – 100.00]	88.57 (21.52) – 100.00 [20.00 – 100.00]	88.57 (21.52) – 100.00 [20.00 - 100.00]	$\chi^2_F(2)$ .50	0.71 (1.82) – 0.00 [0.00 – 5.00]	0.71 (3.31) – 0.00 [-5.00 – 10.00]
between-group		W_s .45			W_s	.09	.63

LOCF	EG	95.00 (7.64) – 100.00 [80.00 – 100.00]	94.21 (9.32) – 100.00 [70.00 – 100.00]	95.26 (9.05) – 100.00 [70.00 – 100.00]	$\chi^2_F(2)$	.07		-0.79 (3.44) – 0.00 [-15.00 – 0.00]	0.26 (4.24) – 0.00 [-15.00 – 5.00]
	CG	86.11 (20.04) – 95.00 [20.00 – 100.00]	87.22 (19.79) – 95.00 [20.00 – 100.00]	87.22 (19.79) – 95.00 [20.00 – 100.00]	$\chi^2_F(2)$	.23		1.11 (2.74) – 0.00 [0.00 – 10.00]	1.11 (3.66) – 0.00 [-5.00 – 10.00]
between-group		W_s .15					W_s	.05	.92
**FM-UE**									
CC	EG	52.38 (15.16) – 58.50 [15.00 – 66.00]	**53.94 (14.69) – 59.50 [15.00 – 66.00] ***	54.06 (13.46) – 59.00 [16.00 – 66.00]	$\chi^2_F(2)$	.11		1.56 (2.25) – 0.50 [-1.00 – 6.00]	1.69 (5.11) – 1.00 [-8.00 – 13.00]
	CG	53.08 (19.01) – 62.00 [5.00 – 66.00]	52.85 (19.10) – 63.00 [5.00 – 66.00]	53.25 (20.70) – 62.50 [6.00 – 66.00]	$\chi^2_F(2)$	.25		-0.23 (1.09) – 0.00 [-3.00 – 1.00]	0.15 (2.73) – 1.00 [-6.00 – 4.00]
between-group		W_s .71					W_s	**.03**	.43
LOCF	EG	53.79 (14.36) – 60.00 [15.00 – 66.00]	**55.11 (13.82) – 60.00 [15.00 – 66.00] ***	55.21 (12.73) – 60.00 [16.00 – 66.00]	$\chi^2_F(2)$	.11		1.32 (2.14) – 0.00 [-1.00 – 6.00]	1.42 (4.71) – 0.00 [-8.00 – 13.00]
	CG	50.44 (19.45) – 62.00 [5.00 – 66.00]	50.25 (19.50) – 60.50 [5.00 – 66.00]	50.56 (20.10) – 60.00 [6.00 – 66.00]	$\chi^2_F(2)$	.25		-0.19 (0.98) – 0.00 [-3.00 – 1.00]	0.13 (2.45) – 0.00 [-6.00 – 4.00]
between-group		W_s .74					W_s	**.03**	.48
**MMSE**									
CC	EG	27.19 (2.20) – 27.50 [23.00 – 30.00]	27.50 (1.46) – 27.50 [25.00 – 30.00]	27.44 (2.06) – 28.00 [22.00 – 30.00]	$\chi^2_F(2)$	.67		0.31 (1.49) – 1.00 [-2.00 – 2.00]	0.25 (1.29) – 0.00 [-2.00 – 3.00]
	CG	27.07 (1.82) – 27.50 [24.00 – 29.00]	27.62 (2.36) – 28.00 [24.00 – 30.00]	**28.14 (1.99) – 28.50 [24.00 – 30.00] ****	$\chi^2_F(2)$	**.03**		0.29 (2.09) – 0.50 [-4.00 – 4.00]	1.07 (1.07) – 1.00 [-1.00 – 3.00]
between-group		W_s .78					W_s	.98	.06

LOCF	EG	27.00 (2.08) – 27.00 [23.00 – 30.00]	26.74 (2.79) – 27.00 [17.00 – 30.00]	26.68 (3.07) – 28.00 [17.00 – 30.00]	$\chi^2_F(2)$	.85	-0.26 (2.73) – 0.00 [-10.00 – 2.00]	-0.32 (2.63) – 0.00 [-10.00 – 3.00]
	CG	26.68 (2.31) – 27.00 [22.00 – 29.00]	27.05 (2.84) – 28.00 [22.00 – 30.00]	**27.63 (2.65) – 28.00 [22.00 – 30.00] ** **	$\chi^2_F(2)$	**.02**	0.37 (1.89) – 0.00 [-4.00 - 4.00]	0.95 (1.13) – 1.00 [-1.00 – 3.00]
between-group		W_s .79			W_s		.77	**.04**

For change over time, we used Friedman's ANOVA test statistic, for within-group post hoc analysis of the differences Wilcoxon's sign rank test, and between-group Wilcoxon's rank-sum test. Significant comparisons with respect to baseline are indicated with * for p-values < .05 and ** for p-values < .01. *BI* Barthel Index, *CC* complete case analysis, *CG* control group, *EF* executive functioning, *EG* experimental group, *FM-UE* Fugl-Meyer Assessment for the upper limb, *LOCF* last observation carried forward, *MMSE* Mini-Mental State Examination, *MoCA* Montreal Cognitive Assessment, *SA* spatial awareness, χ^2_F Friedman's ANOVA test statistic, *T* Wilcoxon's sign rank test, *Ws* Wilcoxon's rank-sum test

Table S4. Improvement or deterioration in ASCS from baseline to after treatment split by different cut-offs as a percentage of total patients (n=30) per group.

Group	Attention		Memory		EF		SA		GCF	
Improvement	*>0.5 SD*	*>1 SD*	*>0.5 SD*	*>1 SD*	*>0.5 SD*	*>1 SD*	*>0.5 SD*	*>1 SD*	*>0.5 SD*	*>1 SD*
EG	13.33%	0	13.33%	3.33%	13.33%	0	30.00%	13.33%	23.33%	6.67%
CG	13.33%	0	10.00%	0	16.67%	0	16.67%	10.00%	13.33%	3.33%
Deterioration	*<-0.5 SD*	*<-1 SD*	*<-0.5 SD*	*<-1 SD*	*<-0.5 SD*	*<-1 SD*	*<-0.5 SD*	*<-1 SD*	*<-0.5 SD*	*<-1 SD*
EG	0	0	6.67%	0	3.33%	3.33%	3.33%	3.33%	3.33%	3.33%
CG	3.33%	0	0	0	6.67%	0	10.00%	0	3.33%	0

CG control group, *EG* experimental group, *EF* executive functioning, *GCF* generalized cognitive functioning, *SA* spatial awareness

Table S5. Improvement or deterioration in ASCS from baseline to follow-up split by different cut-offs as a percentage of total patients (n=30) per group.

Group	Attention		Memory		EF		SA		GCF	
Improvement	*>0.5 SD*	*>1 SD*	*>0.5 SD*	*>1 SD*	*>0.5 SD*	*>1 SD*	*>0.5 SD*	*>1 SD*	*>0.5 SD*	*>1 SD*
EG	20.00%	3.33%	26.67%	3.33%	13.33%	6.67%	33.33%	20.00%	33.33%	10.00%
CG	10.00%	0	16.67%	6.67%	10.00%	3.33%	16.67%	13.33%	20.00%	0
Deterioration	*<-0.5 SD*	*<-1 SD*	*<-0.5 SD*	*<-1 SD*	*<-0.5 SD*	*<-1 SD*	*<-0.5 SD*	*<-1 SD*	*<-0.5 SD*	*<-1 SD*
EG	0	0	3.33%	0	6.67%	0	3.33%	0	0	0
CG	0	0	0	0	3.33%	3.33%	10.00%	6.67%	3.33%	3.33%

CG control group, *EG* experimental group, *EF* executive functioning, *GCF* generalized cognitive functioning, *SA* spatial awareness

REFERENCES

1. Supervia A, Aranda D, Angel Marquez M, Aguirre A, Skaf E, Gutierrez J. Predicting length of hospitalisation of elderly patients, using the Barthel Index. Age Ageing. 2008;37(3):336–9.

2. Peña-Casanova J, Quiñones-Úbeda S, Quintana-Aparicio M, Aguilar M, Badenes D, Molinuevo JL, et al. Spanish multicenter normative studies (NEURONORMA project): Norms for verbal Span, visuospatial Span, letter and number sequencing, trail making test, and symbol digit modalities test. Arch Clin Neuropsychol. 2009;24(4):321–41.

3. Appollonio I, Leone M, Isella V, Piamarta F, Consoli T, Villa ML, et al. The frontal assessment battery (FAB): Normative values in an Italian population sample. Neurol Sci. 2005;26(2):108–16.

4. Woodbury ML, Velozo CA, Richards LG, Duncan PW. Rasch analysis staging methodology to classify upper extremity movement impairment after stroke. Arch Phys Med Rehabil [Internet]. Elsevier; 2013;94(8):1527–33. Available from: http://dx.doi.org/10.1016/j.apmr.2013.03.007

5. Friedman L, Speechley SDM, Teasell R. Evaluating the Montreal Cognitive Assessment (MoCA) and the Mini Mental State Exam (MMSE) for Cognitive Impairment Post Stroke: A Validation Study against the Cognistat Graduate Program in Epidemiology and Biostatistics. 2012;(September). Available from: http://ir.lib.uwo.ca/etd

6. Van der Elst W, van Boxtel MPJ, van Breukelen GJP, Jolles J. Rey's verbal learning test: normative data for 1855 healthy participants aged 24-81 years and the influence of age, sex, education, and mode of presentation. J Int Neuropsychol Soc. 2005;11(3):290–302.

7. Raspelli S, Pallavicini F, Carelli L, Morganti F, Pedroli E, Cipresso P, et al. Validating the Neuro VR-Based Virtual Version of the Multiple Errands Test: Preliminary Results. Presence Teleoperators Virtual Environ [Internet]. 2012;21(1):31–42. Available from: http://www.mitpressjournals.org/doi/abs/10.1162/PRES_a_00077

8. Desmond DW, Moroney JT, Sano M, Stern Y. Recovery of Cognitive Function After Stroke. Stroke [Internet]. 1996;27(10):1798–803. Available from: http://www.pubmedcentral.nih.gov/articlerender.fcgi?artid=3543398&tool=pmcentrez&rendertype=abstract

9. Rasquin SMC, Verhey FRJ, Lousberg R, Winkens I, Lodder J. Vascular cognitive disorders: Memory, mental speed and cognitive flexibility after stroke. J Neurol Sci. 2002;203–204:115–9.

10. van Zandvoort MJE, Kessels RPC, Nys GMS, de Haan EHF, Kappelle LJ. Early neuropsychological evaluation in patients with ischaemic stroke provides valid information. Clin Neurol Neurosurg. 2005;107(5):385–92.

11. Lopez OL, Becker JT, Jagust WJ, Fitzpatrick A, Carlson MC, DeKosky ST, et al. Neuropsychological characteristics of mild cognitive impairment subgroups. J Neurol Neurosurg Psychiatry [Internet]. 2006;77(2):159–65. Available from: http://www.pubmedcentral.nih.gov/articlerender.fcgi?artid=2077558&tool=pmcentrez&rendertype=abstract

12. O'Callaghan CA. OxMaR: Open Source Free Software for Online Minimization and Randomization for Clinical Trials. PLoS One. 2014;9(10):1–10.
